# A multiproxy sediment-core record of lake-level change in paleolake Makgadikgadi (82–21 ka) with implications for human occupation

**DOI:** 10.1038/s41598-026-50559-2

**Published:** 2026-05-18

**Authors:** Julie Lattaud, Sallie Burrough, Ella Walsh, Josh Allin, Joy S. Singarayer, David S. G. Thomas, Negar Haghipour, Moruti Ntloedibe, Godfrey Nkala, Chris Mpelege, Cindy De Jonge

**Affiliations:** 1 Geological Institute, Department of Earth Sciences, ETHZ, Zurich, Switzerland; 2 Department of Environmental Science, Stockholm University, Stockholm, Sweden; 3 School of Geography and the Environment, University of Oxford, Oxford, UK; 4 SFF Centre for Early Sapiens Behaviour (SapienCE), University of Bergen, Bergen, Norway; 5 NORCE Norwegian Research Centre, Bergen, Norway; 6 Department of Meteorology, University of Reading, Reading, UK; 7 School of Geography, Archaeology and Environmental Studies, University of the Witwatersrand, Johannesburg, South Africa; 8 Laboratory of Ion Beam Physics, Department of Physics, ETHZ, Zurich, Switzerland; 9 Botswana Geoscience Institute, Lobatse, Botswana; 10 Botswana Ash, Sowa, Botswana

**Keywords:** Paleolake Makgadikgadi, Biomarker, OSL, Kalahari, Middle Stone Age, Climate sciences, Ecology, Ecology, Environmental sciences, Solid Earth sciences

## Abstract

The Makgadikgadi Basin in central Botswana is a key region for understanding Stone Age archaeology and human evolution. However, paleoenvironmental reconstructions have so far offered only fragmentary insights into past hydroclimatic and environmental conditions. We present the first environmental record from Sowa Pan, paleolake Makgadikgadi, covering the last 82–20 ka (Marine Isotope Stages 5–2). Sedimentological and biomarker lipid analyses reveal four distinct lake phases, reflecting major hydrological shifts and sediment provenance. From 82 to 75 ka, the lake was shallow, with lowest lake levels temporally coinciding with Middle Stone Age archaeological material (silcrete lithics, mainly unifacial and bifacial points) on the western lakebed. Between 75 –58 ka, the lake refilled, likely fed by river inflow sourced from the Angolan highlands. A second low stand likely occurred around 58–56 ka. From 52 to 37 ka, geochemical proxies indicating a change in lake inflow with less influence of the Kwando catchment. During this time, the lake had high primary productivity and biomarkers for terrestrial herbivores are recorded in the sediments. After an accumulatory hiatus, the youngest unit (ca. 21 ka) marks the formation of the Sowa Spit, characterized by high sand input and minimal organic preservation. This record refines the chronology of lake-level changes and reveals that desiccation phases provided ecologically viable landscapes for human activity. It also identifies shifting sediment and nutrient sources that shaped lake dynamics during the late Pleistocene.

**Supplementary Information:**

The online version contains supplementary material available at 10.1038/s41598-026-50559-2.

## Introduction

**Fig. 1:**
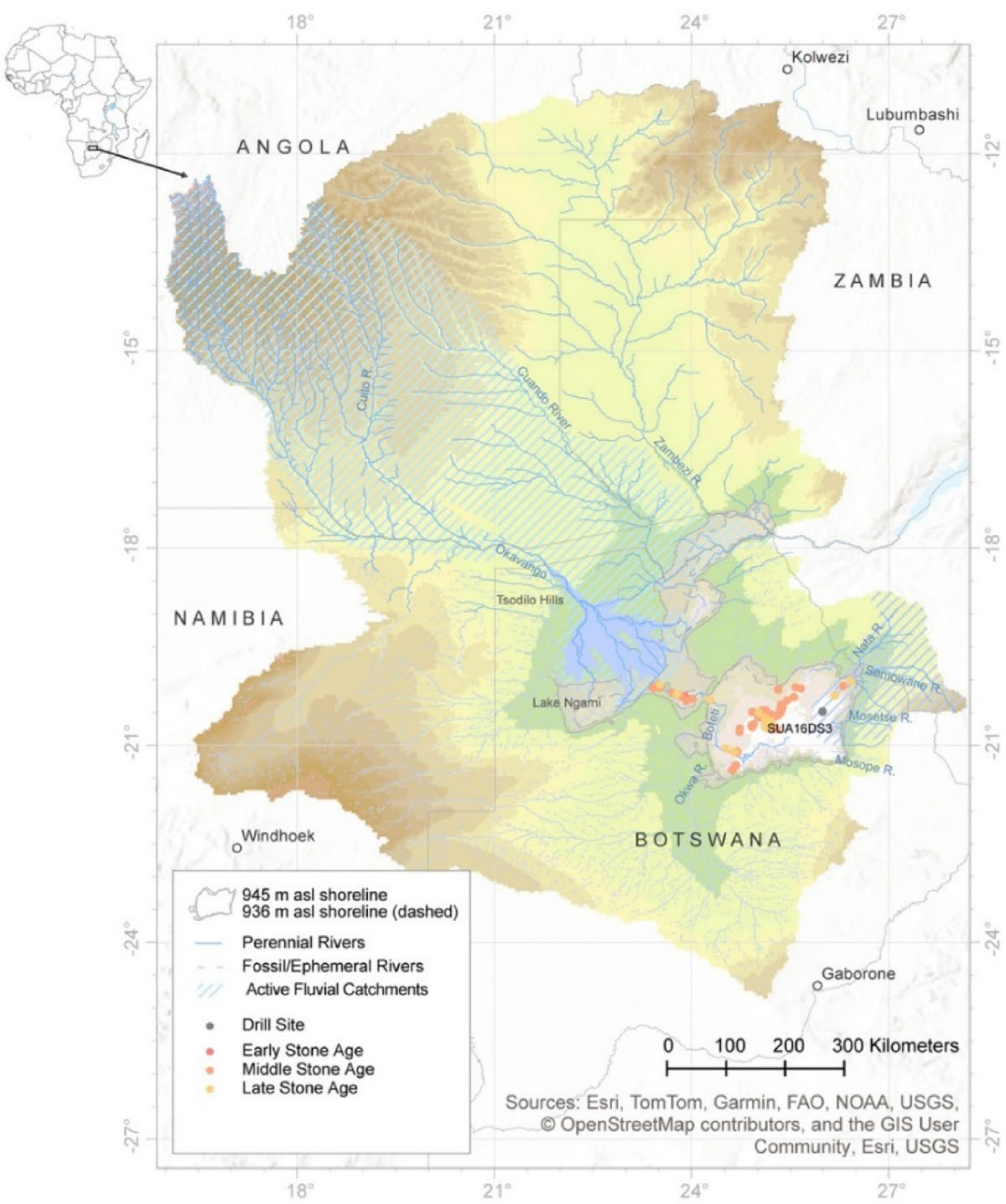
Core SUA16DS3 location and hydrology of the catchment. The archaeological sites from the Late, Middle and Early Stone Age are highlighted from^21^. Map created in *ArcGIS Pro*, Version 3.6.0^104^.

The Makgadikgadi Basin in central Botswana is of major human evolutionary significance^1^ and has sparked debate in anthropology^2^. Some mitogenomic studies go so far as to suggest, highly controversially^3–5^, that it might be a cradle of modern humans, with genetic evidence dating back 200 ka^6^. Today, the basin is dominated by extensive salt pans, principally Ntwetwe (~ 9,000 km²) and Sua (Sowa; ~3,200 km²), together forming one of the largest playa complexes in the world. During the Pleistocene, however, the basin hosted a vast lacustrine system, paleolake Makgadikgadi, characterised by a mosaic of interconnected geomorphic environments including shoreline zones, fluvial inflow corridors, deltaic and marginal wetlands, creating spatially and temporally variable freshwater to brackish habitats^7–17^. More than 200 Middle Stone Age (MSA, ca. 280–45 ka BP^18^) sites exist here, with those that have been excavated suggesting occupation during a period of very low or dry lacustrine conditions when the basin floor provided resources, especially for silcrete tool manufacture^19–25^ (Fig. 1).

Two main models debate the lake’s history^20,26^: the Quiescent model^27,28^ suggests a permanent shrinkage around 500 ka due to tectonic changes diverting inflows eastward, while the Dynamic model^20^ argues for fluctuating water levels with intermittent lake desiccation during the late Quaternary. Testing these models has been hindered by limited site access, poor preservation, and dating challenges. Optically stimulated luminescence (OSL) ages of lake paleo-shorelines show lake presence at intervals between 105 and 8.5 ka^8,20^, but only reveal high stands, not dry phases^29,30^. Other regional studies indicate a fluctuating hydroclimate both spatially in Botswana and temporally during the last 80 ka^31–35^.

Under present-day conditions, Sowa Pan is fed primarily by the seasonally-flowing Nata River, which drains the Zimbabwean Plateau from the northeast, together with smaller ephemeral rivers (including the Semowane, Mosetse, Lepashe and Mosope) draining the low-relief uplands of eastern Botswana (Fig. 1). The wider Makgadikgadi Basin, however, received additional inflows in the past. These likely included substantially stronger contributions from the Angolan Highlands via the Okavango–Kwando system, which fed the Boteti River entering western Makgadikgadi and which occupies an oversized valley relative to its present-day discharge^14^ (Fig. 1). Late Quaternary diatomaceous deposits^10,16^ indicate that at times this inflow was sufficient to cause ponding in the Makalamabedi region, upstream of the basin margin, forming an extensive, shallow water body (Fig. 1). Along the southern basin margin, presently dry river valleys such as the Okwa and Letlhakane have also been identified as potentially important past surface inflows. Although these southern systems remain poorly constrained, a single radiocarbon age on freshwater gastropods indicates flowing water in the Okwa around 17 ka^12,36^.

This study focuses on Sowa Pan, located in the eastern Makgadikgadi basin (Fig. 1), which represents the lowest lying and hydrologically most persistent sector of paleolake Makgadikgadi, with the greatest preservation potential for accumulated lake sediments. The aim was to refine the timing of lake low- and high-stands during Marine Isotope Stages (MIS) 5–2 (82–21 ka), assess lake productivity, and constrain sediment provenance through integrated sedimentological and geochemical analyses of lacustrine deposits. Importantly, relatively modest increases in lake level would have resulted in substantial basin-wide connectivity. Lake levels exceeding ~ 908 m above sea level (approximately 3 m above the modern basin floor) would have hydraulically connected the Sowa and Ntwetwe pans, integrating much of the central Makgadikgadi Basin into a single water body. Under such conditions, sedimentation at Sowa Pan would reflect basin-scale hydrological states rather than purely local processes.

## Results

### Core description and age model

Core SUA16DS3 (320–720 cm below land surface, bls) contains four sediment units, differentiated by grain size and XRF ratios (SI Appendix, Fig. S1a). For the following results and discussion, depth is given from the top of the core and not bls. Sandy Unit I (0–44 cm) (> 63 μm, 74 ± 17%), silty Unit II (44–207 cm) (2–63 μm, 86 ± 7%), relatively clay-enriched Unit III (207–277 cm) (< 2 μm, 19 ± 5%), and silty Unit IV (279–372 cm) (90 ± 3%). The Bayesian age model, based on 19 OSL dates (8 from the short SUADS1 cores above SUA16DS3 and 11 from SUA16DS3, see Methods section), indicates that the core spans 82 ± 8 to 20 ± 1.8 ka, with an apparent chronological hiatus from 34.6 to 21.3 ka (SI Appendix, Fig. S1b). Units correspond to MIS 2 to MIS 5a respectively. Sedimentation rates vary from 0.007 cm yr^− 1^ below the hiatus to 0.05 cm yr^− 1^ above.

**Fig. 2:**
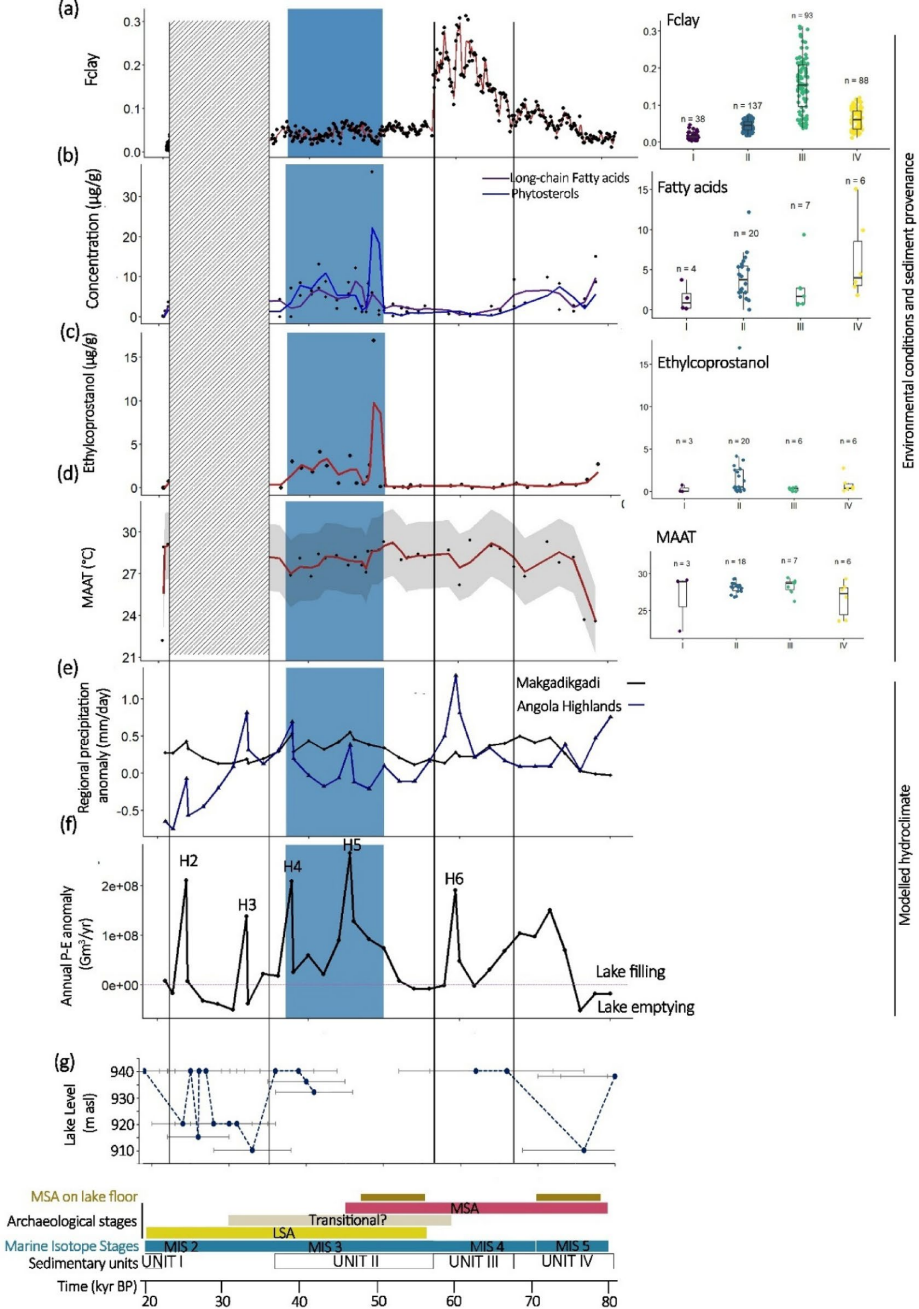
Proxies indicative of environmental conditions, for each proxy a downcore record (the line represent the 2-point average) and a boxplot average and distribution of the data per sedimentary unit are presented. (**a**) relative clay content, (**b**) long-chain fatty acids and phytosterols concentration, (**c**) 24-ethylcoprostanol concentration, (**d**) reconstructed mean annual air temperature (MAAT) from the African lakes calibration^37^, as well as modelled hydrological conditions (**e**) modelled precipitation anomaly for the Angola Highlands (16.875–20.625 °E, 16.25–11.25 °S) and the Makgadikgadi region (20.625–28.125 °E, 21.25–16.25°S), (**f**) modelled annual precipitation-evaporation (P-E) anomaly bias corrected using the ERA5 modern reanalysis for the region, (**g**) reconstructed lake level from^20^. Timing of the archaeological periods within the wider Kalahari basin (bottom) and lake floor MSA tools are from^20^. Striped area indicates the sedimentary hiatus and the blue shaded area the potential anoxia period.

### Proxies for sediment provenance and watershed conditions

Grain size and elemental XRF log-ratios are used to reconstruct varying sediment sources (Fig. 2, Figs. S1a, S2). Grain-size variability in the Makgadikgadi record primarily reflects changes in water depth, hydrodynamic energy, and water-column residence time rather than sediment supply alone^38,39^. Clay-sized particles accumulate preferentially under deep, stable lacustrine conditions with limited resuspension, whereas under shallow or dynamic lake and pan settings clay settling is inhibited as particles remain in suspension or are laterally redistributed within the basin. Silt-sized particles, by contrast, can settle episodically even under moderately energetic conditions. Grain-size distributions are therefore a first-order indicator of relative lake depth and stability at the coring site. Unit I shows sandy sediments enriched in Si, and Zr associated with the spit formation, and Unit III a strong percentage of fine-grained clay sediments enriched in K, Rb and Ti (Fig. S2), potentially representing highly weathered input into the lake and/or a phase when smaller grain size particles such as clay can settle into the lake undisturbed. Both Unit II and IV have high silts with low clay content while Unit IV is enriched in Cl and Ru (Fig. S2).

Long-chain fatty acids and phytosterols, used to reconstruct changes in vegetation-derived organic matter^40^ (Table 2), show decreasing concentrations (normalized over g sediment) over time in Unit IV, very low concentrations in Unit III and peaks during Unit II (Fig. 2b). They are below detection limit in Unit I. Herbivore biomarkers (24-ethylcoprostanol) appear only in Unit II (~ 37–49 ka) (Fig. 2c).

**Table 1:** Biomarkers used in this study, their likely source organisms in terrestrial environments and their environmental diagnostic.

Biomarker	Systematic name	Source organism	Environmental indication	References
Long-chain fatty acids (nC > 24)		Terrestrial plants	Terrestrial plants present in the catchment, wetter climate	^61^
Branched GDGT		Soil or aquatic Bacteria	Specific pH and temperature conditions	^105,106^
Long-chain diols		Eustigmatophyte	Primary production	^51,107,108^
Phytosterols (β-sitosterol, campesterol)	24-ethylcholest-5-en-3β-ol 24-methylcholest-5-en-3β-ol	Mainly terrestrial plants, minor algae and cyanobacteria	Plant productivity	^109,110^
24-ethylcoprostranol	24-ethyl-5β-cholestan-3α-ol	Mammal faeces	Presence of herbivorous mammals	^111,112^
Tetrahymanol	Gammaceran-3β-ol	Freshwater ciliate in the absence of oxygen	Likely water column stratification	^45,113,114^

**Table 2:** Equations used for environmental parameter reconstruction.

Ratio	Parameter	Equation	Reference
$\:CB{T}^{{\prime\:}}=\mathrm{log}\frac{(Ic+II{c}^{{\prime\:}}+II{b}^{{\prime\:}}+II{a}^{{\prime\:}}+III{c}^{{\prime\:}}+III{b}^{{\prime\:}}+IIIa{\prime\:}}{(Ia+IIa+IIIa)}$	pH	pH = 8.95 + 2.65 x CBT’	^37^
$\:MB{T}^{{\prime\:}}5ME=\frac{(Ia+Ib+Ic)}{(Ia+Ib+Ic+IIa+IIb+IIc+IIIa+IIIb+IIIc)}$	Air temperature (MAAT)	MAAT=−1.21 + 32.42 x MBT’5ME	^37^
$IR^{\prime}6 + 7ME = \frac{{\left( {\left( {IIa^{\prime} + IIb^{\prime} + IIc^{\prime} + IIIa^{\prime} + IIIb^{\prime} + IIIc^{\prime}} \right) \times 0.5 + IIIa^{\prime\prime\prime} + IIa'''} \right)}}{{\left( {IIa + IIb + IIc + IIIa + IIIb + IIIc + IIa^{\prime} + IIb^{\prime} + IIc^{\prime} + IIIa^{\prime} + IIIb^{\prime} + IIIc^{\prime} + IIIa^{\prime\prime\prime} + IIa^{\prime\prime\prime}} \right)}}$	Salinity		^55^
$Paq = \frac{{C22 + C24}}{{C22 + C24 + C26 + C28 + C30 + C32}}$	Submerged macrophyte productivity	Higher when more macrophyte	^52,61^

**Fig. 3:**
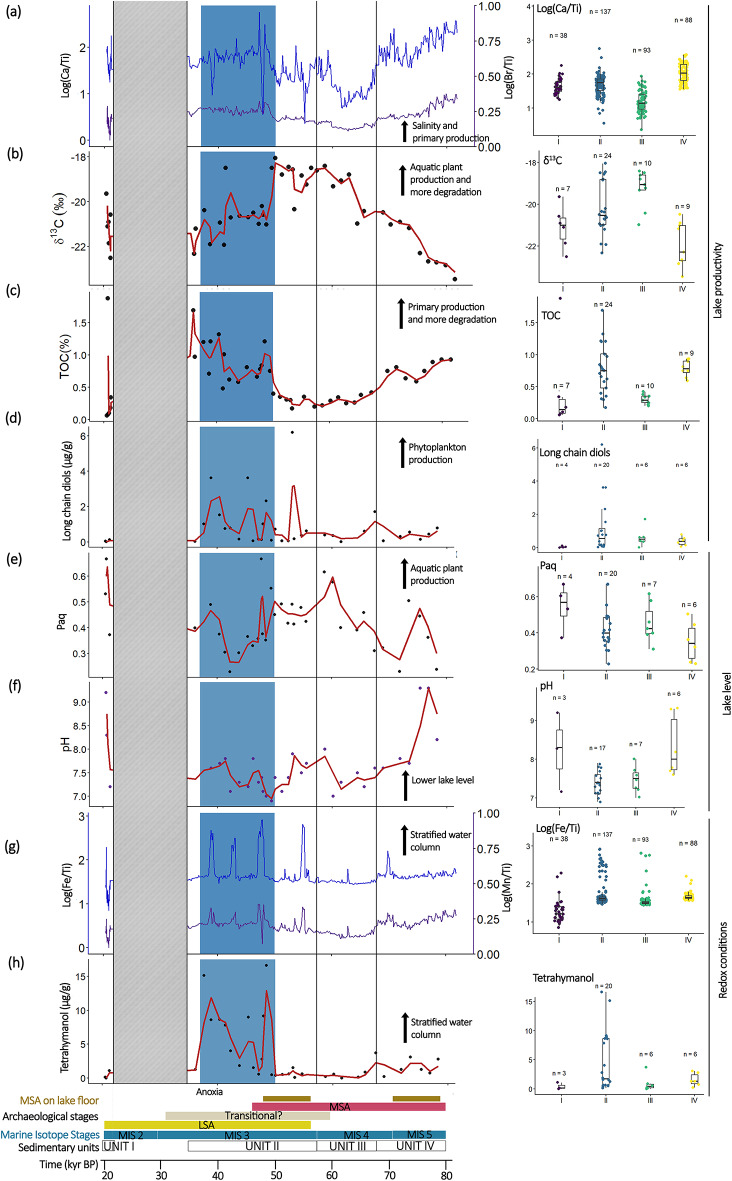
Proxies indicative of in situ lake conditions, for each proxy a downcore record (the line represent the 2-point average) and a boxplot average and distribution of the data per sedimentary unit are presented. (**a**) log(Ca/Ti) and log(Br/Ti), (**b**) δ^13^C (‰ vs. VPDB), (**c**) TOC (%), (**d**) long-chain diol concentration, (**e**) Paq, (**f**) reconstructed pH, (**g**) log(Fe/Ti) and log(Mn/Ti), (**h**) tetrahymanol concentration. Timing of the archaeological stages and lake floor tools are from^20^. Striped area indicates the sedimentary hiatus and the blue shaded area the potential anoxia period.

### Proxies for in situ lake conditions (productivity and preservation)

Ca/Ti-normalized log-ratio correlates with log(Sr/Ti) (*r* = 0.75, *p* < 0.01) indicating that Ca is likely derived predominantly from the lake itself (with co-precipitation of SrCO_3_^41^). Log(Ca/Ti) was high during Unit IV, lowest during Unit III, during Unit II and I it increased at the beginning of the unit before stabilizing (Fig. 3a, Fig. S1a). Log(Br/Ti) follows a similar pattern (Fig. 3a). In this inland system these ratios are likely indicative of changing salinity, also due to their correlation with log(Cl/Ti) (Fig. S1a).

The organic carbon stable isotope signature (δ^13^C) of the sediment is close to −23‰ at the bottom of Unit IV and slowly increases, reaching a plateau at −19‰ during Unit III (Fig. 3b). Units II and I have a decreasing δ^13^C down to −22‰ (average of −21.9 ± 0.9‰). Total organic carbon (TOC) is in general below 1% and shows a decreasing trend in Unit IV, with an all-time low in Unit III and I (TOC < 0.5%) (Fig. 3c). Unit II presents periods of relatively enhanced TOC content with a constant increase until the hiatus (48 ± 4.4–35 ± 4.4 ka, TOC > 0.5%).

Redox-sensitive elements (Fe and Mn)^42^, show four sharp increasing events during Unit II (Fig. 3g). One of the events is located at the top of subcore PC2 (55 ± 4.4 ka, corresponding to the bottom of the top core PC1 and the top of the second core PC2, see Methods section), indicating that oxidation of the sediment could have occurred during coring due to corrosion by the steel tube or after coring during storage. The other increases (peaks at 38 ± 4.4, 43 ± 4.4 and 47.5 ± 4.4 ka) could be indicative of a stratified water column, and/or anoxic bottom water, and/or a change in sediment source^43^. For the latter, a change in grain size or a relation with detrital element such as Ti, Rb and K would be expected, which is not observed (Fig. 2a, Fig. S2b). The log-ratios of Fe and Mn are correlated, although weakly, with log(S/Ti) (*r* = 0.48, *r* = 0.35, *p* < 0.01) which increases for the two older events (43 ± 4.4 and 47.5 ± 4.4 ka), which can be indicative of the deposition of FeS, in agreement with potential sedimentary anoxic conditions^44^. In addition, tetrahymanol, a lipid produced by ciliates during water column stratification event, also peaked during Unit II (49 ± 4.4 and 36 ± 4.4 ka, average concentration of 6.7 µg/g, Fig. 3h). During stressing conditions such as anoxia in the water column, ciliates will switch diet, feeding on bacteria at the oxic/anoxic boundary and synthesize tetrahymanol^45^. Both inorganic (Fe and Mn log-ratios) and biomarkers are thus indicating a stratified water column with potential anoxia during Unit II.

Long-chain diols are produced in lakes by unicellular algae Eustigmatophyte^46–48^ (Table 2), and shifts in the isomer proportions can be linked to lake surface temperature, nutrients concentration and stratification^47–51^, while their abundance can be used to represent lake primary productivity as they are produced by photosynthetic algae^48^. Their sedimentary distribution showed a dominant C_32_ 1,15-diol, in agreement with a production by Eustigmatophytes (Fig. S3b). The total concentration of the isomers is low in Unit IV, showed a small increase at the Unit IV/III boundary then decreasing with time to values that were almost below detection limits in Unit III (Fig. 3d). Their concentration increased significantly in Unit II with several peaks whereas they are below detection limits in Unit I (Fig. 3d). The isomer proportion was constant in all units but Unit II where the C_30_ 1,15-diol became dominant (Fig. S3b). This shift occurred during a period of stratified water column and potential anoxia (52 − 37 ka) and aligns with existing interpretations that elevated C_30_ 1,15-diol abundances in lakes are associated with a change in nutrients or decreased temperature^47,51^, and decreased C_32_ 1,15-diol with a decrease in oxygenation of the lake^51^.

Paq values, indicative of macrophyte production in lake systems^52^ (Table 2), vary between 0.22 and 0.61 (Fig. 3e). The ratio is high in Unit IV and at the boundary of Unit III/II, and decreases during Unit II and I.

Branched Glycerol Dialkyl Glycerol Tetraethers (brGDGT), bacterial membrane lipids^53^, have dominant brGDGT-III (46 ± 11%, Fig. S3c) which is indicative of in situ lacustrine bacterial production. The presence of the 7-methyl isomer and late eluting isomers (here named brGDGT-IIIa^α^, -IIIa^β^ and -IIa^α^, -IIa^β^)^54^ is indicative of a saline environment^55^. Their relative distribution does not change downcore except at the onset of the potential anoxia period (~ 50 ka) where IR_6ME_ (the ratio of the 6- over the 5-methyl brGDGTs) decreased (0.78 to 0.65, Fig. S3d). As no major change in brGDGT source (e.g., soil versus aquatic) is identified using the ternary plot (Fig. S3c), mean annual air temperature (MAAT) can be reconstructed using only the 5-methyl brGDGT (MBT’_5ME_) and the African lake calibration^37^ (Table 2). Reconstructed MAAT varied little after an initial increase during Unit IV from 22 to 28 °C (calibration error is ± 2.4 °C^37^, Fig. 2d). Modern air temperatures in the Makgadikgadi Basin varies between 17 °C and 28°C^56^ (1985–2014 average).

Similarly to MAAT, pH can be reconstructed using the brGDGT-based CBT’ index^37^ (Table 2). Reconstructed pH shows alkaline water (pH > 8) in Unit IV and I, whereas there was little change during the rest of the record (averaging around 7.5, Fig. 3e). Modern pH in the Sowa pan during flooding is high, with values between 8.6 and 10^57,58^, while modern pH in the Okavango Delta panhandle varies between 6 and 7.4^59^.

### Climate model

Climate model outputs from HadCM3 (Fig. 2e,f) suggest decreased precipitation in the early part of Unit IV (80 − 60 ka) both locally and in the Angolan Highlands part of the northern catchment. This is followed by a strong increase in local precipitation around the Makgadikgadi basin and high P-E anomaly at the end of Unit IV indicating lake filling. Unit III is characterized by a short-term increase in precipitation in the Angolan Highlands around 60 ka driven by a simulated Heinrich event 6, H6^60^. Unit II has a modelled precipitation increase locally and a peak in the Angola Highlands during H5, whereas Unit I showed a strong decrease in the Angolan Highlands precipitation in the model following H2. Both headwater (Angola Highlands) and distal (Makgadikgadi) ends of the catchment predict an increase in precipitation in response to modelled Heinrich events, partly as a result of a southerly shift in the tropical rainbelt. However, they respond almost anti-phase to multi-millennial orbital forcing. For example, around 70 ka, just before the transition to Unit III, rainfall over the Makgadikgadi basin is at a local maximum, while Angola Highlands rainfall, ca.1200 km to the northwest (Fig. 1), is at a local minimum. The same is seen around the middle of Unit II, although the presence of H5 complicates the response.

The resultant P-E (Fig. 2f) in the early part of Unit IV is therefore predicted to be negative and would likely have resulted in lake shrinkage or desiccation. This period coincides with MSA tools on the lake floor^4,20,21^. Negative P-E over the modelled catchment is also simulated at the transition from Unit III to Unit II, and in much of Unit I. These would be periods when the climate model simulations suggest the lake would be primed for desiccation, although hydrological modelling would be needed to investigate this further. Sharp increases in P-E are seen in the model in response to Heinrich event forcing. As stated above, the catchment as whole experiences an increase in rainfall at these points, as opposed to spatially variable responses to orbital forcing. This results in rapid and short-lived positive changes in the modelled surface water balance.

## Discussion

The sedimentary record from paleolake Makgadikgadi reveals a dynamic lacustrine system with marked fluctuations in hydrology and productivity spanning MIS 5a (Unit IV) to 2 (Unit I). The data delineate four distinct lake phases, each reflecting shifts in climate, catchment hydrology, and ecosystem response.

### Unit IV (MIS 5a, ca. 82–67 ka): a shallow lake

The sedimentology and geochemistry suggest a shallow lake. The dominance of silt with low clay content in Unit IV indicates limited water-column stability and frequent reworking during a shallow lake phase. Increasing δ¹³C values can originate from three processes: (i) a shift in dominant primary producers from phytoplankton to macrophytes, (ii) increased bacterial degradation and (iii) a decreased use of bicarbonate relative to pCO_2_. The first of these (i) is supported by an increase in macrophyte indicators (Paq, Fig. 3e) and reflects similar variations in the modern Okavango Delta where flooded areas record lower δ¹³C values in comparison to seasonally dry areas^61^. In the second of these scenarios (ii) the recorded δ¹³C shift is too large (2–3‰) to be solely explained by a change in bacterial degradation^62^. In the third (iii) it is notable that the reconstructed pH during Unit IV is decreasing (Fig. 3f) providing more HCO_3_^−^ and pCO_2_ which should promote lower δ¹³C values in phytoplankton^63^. This third scenario is unlikely to be the dominant process responsible for increasing δ¹³C values. Both (i) and (ii) are consistent with a low water-level lake where the coring site is located close to the paleo-edge of the lake. Elevated log(Br/Ti), log(Ca/Ti), and log(Cl/Ti) imply increased salinity, likely linked to lower water levels (Fig. 3a, Fig. S1a). Elevated reconstructed MAAT at 75–73 ± 8 ka (+ 3 °C, Fig. 2d) suggesting high evaporation, further supports a shallow lake phase. Leaf waxes and phytosterols, albeit low in concentration, indicate a low, but detectable terrestrial (dust or river) input into the lake (Fig. 2b). Hence there was adequate input of terrestrial organic matter into the lake to record a signal from the catchment. In addition, the detection of tetrahymanol, points to stratified water column conditions, although log(Fe/Ti) remained low throughout the unit (Fig. 3g,h). The modelled negative P-E balance in the region (Fig. 2f) is consistent with the palaeodata interpretation of a contracting lake. The negative P-E aligns with modelled decreases in precipitation affecting the Angolan Highlands (Fig. 2e) and minimum insolation at 15°S indicating some influence of orbital forcing on rainfall in Makgadikgadi^64^. Overall, the implication is that during this time paleolake Makgadikgadi was very low, with increased macrophyte production (Fig. 3e), in agreement with low lake-level reconstructed with shoreline dating^20^ (Fig. 2g). Refitted MSA archaeology from Ntwetwe pan demonstrates that stone tools were made in-situ on the pan floor during this time, from silcretes exposed within the lake basin itself^19,22^. The interpretation is that parts of the lake bed were at least seasonally desiccated and that silcrete was readily exposed during this time^20^.

### Unit III (MIS 4, ca. 67–56 ka): a variable lake level, shift in water supply and low productivity

A marked increase in clay content at the beginning of this unit (Fig. 2a) and sedimentary proxies indicative of terrestrial clastic input (decreased TOC due to dilution by clastic particles, Fig. 3c) denote a rising lake level, likely driven by enhanced rainfall and runoff (Fig. 2f). The increased clay content in Unit III reflects some periods of deeper and more stable lacustrine conditions, allowing sustained settling and accumulation of clay-sized particles at the coring site. Alternatively, a major change in riverine input, for example, flooded rivers entraining clay particles from floodplains upstream of the lake basin, is also possible. However, the age of Unit III matches paleo-shorelines that indicate a high-stand between 66 ± 5 and 62 ± 8 ka^20^ (Fig. 2g), and ages of dated lake sediments in Ntwetwe (Fig. 1), which also cluster at ca.66 ka^20^ indicating a regional wet period. This period follows a peak in HadCM3-modelled precipitation in the Makgadikgadi region around 68–70 ± 8 ka, and overlaps (within age uncertainty) with the precipitation peak in the Angola Highlands around 58–62 ± 8 ka (Fig. 2e) driven by the precessional cycle and modelled H6. The main geochemical composition of the sediments, delivered by riverine inflow, during this period differ from the rest of the record (Fig. S2a). Levels of V (and Pb) are elevated (Fig. S3a), which may originate from vanadiferous (vanadium-rich) deposits common in the Kwando catchment located in the Eastern part of the Angola Highlands^65^, but also found in Eastern Zambia and Zimbabwe^66^. Although data are scarce to geochemically (using XRF ratios and biomarkers) qualify all the catchments^67^ and be certain of the primary source of water, at least part of lake inflow was derived from the Angolan Highlands through the Okavango Delta and the Boteti. A deeper study on clay mineralogy and neodymium/strontium ratios^67–69^ are needed to confirm this theory.

Within the lake, the reduction in all biomarker concentrations suggests lower in-lake productivity favouring macrophyte production (evidenced by high Paq values, Fig. 3e). δ¹³C values are increasing towards stable values at −18‰ at the end of the unit (Fig. 3b), also pointing toward dominant input from macrophytes. The large clay input could have changed the turbidity in the lake, making it unsuitable for phytoplankton growth and more favourable for macrophytes. The lake expansion had an impact on the amount of terrestrial organic matter reaching the core site with low phytosterol and fatty acids concentrations (Fig. 2b) recorded during this period. In addition, the large clay input diluted the organic matter and TOC content remained low (Fig. 3c). The decline in lake pH started in Unit IV continued until the middle of Unit III reaching neutral values by 62 ± 8 ka (Fig. 3f). The absence of tetrahymanol and the low log(Fe/Ti) point to a dynamic oxic low-level lake.

Towards the end of the period represented by Unit III, an abrupt shift in grain size at 58 and 56 ka, (Fig. 2a) suggests a major change in sediment provenance and an abrupt cessation of inflows or sedimentation of the clay particles. This event coincides with an insolation minimum at 15°S^64^ which could suggest a second lake dry-out or, at least a marked lake-level fall followed by reflooding. This is supported by negative P-E anomalies indicating a desiccating lake (Fig. 2f) and by the presence of MSA tools found in-situ on the lake bed. While these tools post-date Unit III, bracketing maximum ages on this archaeological material has not yet been established^20^. In addition, no shoreline dates, and independent lake level estimates, are available for this time period (Fig. 2g). The results from this study suggest that the timing of the dry period relating to use of the dry lake bed by MSA humans at this time can be refined to a low-stand that occurred around 58–56 ka (± 6 ka).

### Unit II (MIS 3, ca. 54–37 ka): high stand and productive lake

Unit II is characterised predominantly by silt-sized sediments with limited clay, suggesting a dynamic lacustrine environment with fluctuating water levels, where episodic sediment input and periodic reworking inhibited prolonged clay accumulation despite generally wetter conditions.

This interval is characterized by high TOC content (average of 0.75 ± 0.36%, Fig. 3c), lower δ¹³C values (from − 18 to −21‰, Fig. 3b) and increased biomarker concentrations indicative of elevated primary productivity and organic matter preservation. Paq values decreased (Fig. 3e) indicating a diminished macrophyte input whereas higher concentration of long-chain diols (Fig. 3d) with an increase in the C_30_ 1,15-diol (Fig. S3b) suggest enhanced phytoplankton primary productivity and nutrient input^51^. The first appearance of 24-ethylcoprostanol during this unit (Fig. 2c) signals the presence of large herbivores in the vicinity of the paleo-lake, and the input of phytosterols and long chain fatty acids (Fig. 2b) indicate increased vegetation cover in the lake’s catchment.

Sediment geochemistry (V and Pb counts) indicates a relatively reduced influence from the Kwando catchment (Figs. S2, S3a), suggesting a shift in dominant inflow sources. This is in agreement, within age model uncertainties, with a recent study refining the timing of the capture of the Upper Zambezi system, which includes the Kwando inflow, by the Middle Zambezi and its diversion to the east around 40 ka^70^. There are only a few fossil gastropods (^14^C ages at ca. 45.6 ± 1.2 cal kyrs and 46.4–43.7 cal kyrs BP) for this period^7^, suggestive of fluvial dynamism in inflow channels. With the upper Zambezi system now cut off, this is suggestive of inflow, either episodically or permanently, from the Okavango system in the west, via the Boteti, and the Nata, in the east (Fig. 1). Evidence of a humid MIS 3 also comes from a sediment sequence originating from a small paleo lake adjacent to Tsodilo Hill, north of Makgadikgadi basin^33^, and from the HadCM3 modelled local precipitation which suggests a significantly enhanced P-E anomaly during MIS 3 (Fig. 2f). Despite proxy and model evidence for wetter conditions, there is no direct geomorphological evidence within paleolake Makgadikgadi to support a lake high stand^20^. Elevated lake levels likely periodically restricted access to the lake bed (Fig. 2g), which could have influenced human settlement and stone tool production patterns, which previously were reliant on material sources exposed on the lake floor^4^^21^,. However, the lake seems to have been highly productive, sustaining larger mammals which could make the region a suitable habitat for early humans. LSA sites have been described in and around Makgadikgadi, along the Boteti^71,72^ and Nata^73^ as well as along the eastern and southern shore of Sowa Pan^74,75^ (Fig. 1). North of Lake Makgadikgadi, at Tsodilo Hills and within the wider system on the shore of Lake Ngami (Fig. 1), the early LSA has been dated from ca. 48 ± 5 ka, and in Botswana the record of human occupation, continues almost up to the present day.

### Unit I (MIS 2, ca. 23–21 ka): drought and sand

The sedimentary hiatus between 37 and 23 ka indicates either a prolonged drying phase during which there was very little sediment deposition or a significant erosion event or events. Major loss of sediments via deflation during dry periods is a significant dynamic of the system that continues today^76^. This episode is consistent with regional paleoclimate reconstructions indicating decreased precipitation (Fig. 2e) and lower lake levels^20^ (Fig. 2g). Following this hiatus, the record resumes with increased sand content (74 ± 0.19%) which likely reflects the localized influx of fluvial sands or high local precipitation (Fig. 2e) resulting in the formation of the overlying cuspate spit within a shallow lake system^9^. Specifically, the protrusion of Sowa Spit and other spits along the eastern margin, occurs in association with fluvial inflow points to Sowa pan and are likely the result of strong wave-action and high sediment load following a prolonged dry phase. The formation of such ‘Azov type’ spits has been shown to occur in shallow bodies of water when waves are obliquely incident^77,78^. In agreement, lake productivity proxies are low or under detection limits during this period (Fig. 3). Leaf wax concentrations remain elevated at the onset of Unit I with 4.4 µg g^− 1^ and 0.2 µg g^− 1^ at 21.3 ka (Fig. 2b) indicating low levels of riverine and aeolian inflow that ceases with the formation of the Sowa Spit. These environmental constraints, strong drought and/or flooding would have influenced human use and resource availability in the basin during the Last Glacial Maximum. In the southwest of Makgadikgadi, fossil ungulates and LSA tools are associated with a shallow lake^71^. However, there is currently very little research on the LSA within the basin and no sites with secure chronologies, despite the observed presence of archaeology from this period^21^ (Fig. 1).

Overall lake-level dynamism in paleolake Makgadikgadi seems to have been driven by changes in precipitation amount at a regional level.

### Implications for human evolution and paleoenvironmental dynamics

This study demonstrates the potential of salt pan sediments to yield critical insights into hydrological change in dryland systems. The SUA16DS3 record provides key evidence for the role of hydroclimatic variability in governing basin behaviour within the longer-term context of a progressively diminishing paleolake Makgadikgadi through MIS 5–2. These trends are consistent with tectonically driven reorganisation of inflowing river systems, including independent evidence for capture of the upper Zambezi and its associated tributaries by the lower Zambezi during this interval^70^. The resolution of late-stage lake contraction in this record is, however, constrained by geomorphic context. Emplacement of the Sowa Spit and the associated depositional hiatus limit preservation of sediments from the last ~ 40 ka at this site. As a result, while core SUA16DS3 captures long-term lake behaviour and progressive diminution through MIS 5–2, it does not resolve younger low-stand dynamics in detail. These later phases are better preserved in short vibracores recovered from deeper sump areas of the basin, which provide higher-resolution records of the last ~ 40 ka over shorter time spans (Burrough et al., in prep.).

The complex hydrological variations during the late Pleistocene presented here, with alternating wet and dry phases would have radically shaped ecological landscapes. The lake’s fluctuating extent affected lake floor availability, the location and movement of herbivores and thus the use of the landscape by MSA and LSA humans. As recent geoarchaeological research^4,20,21^ shows, these findings challenge simplistic models portraying drylands solely as ecological barriers, instead highlighting their potential as dynamic, resource-rich environments during periods of low lake stands^4,20,21^. The detailed multiproxy evidence from core SUA16DS3 suggests the window of opportunity for adaptation to, and occupation of, a dry lake bed was surprisingly narrow, with desiccation phases dating to ca. 82 − 67 ka and again at ca. 58 − 56 ka, however the age model errors ~ 8 ka need to be taken into consideration. The results underscore the importance of integrating paleoenvironmental and archaeological data to better understand human-environment interactions in southern Africa’s drylands.

## Materials and methods

### Study site and sampling

The paleolake Makgadikgadi was formed with the activation of the Okavango graben during the Middle to Late Pleistocene in the north-west of the Makgadikgadi Basin that led to the formation of the Okavango Delta and the Makgadikgadi Pans^20^. Paleolake Makgadikgadi has experienced fluctuating hydrological conditions over the last 300,000 years, with periods of high lake stands sustained by inputs from the Upper Zambezi, Boteti, and eastern rivers, as well as groundwater^20,22^. The modern Makgadikgadi Basin hosts the world’s largest salt pan complex, including the two largest pans, Sowa and Ntwetwe (Fig. 1a).

Fieldwork was done in collaboration with the Geosciences Institute of Botswana and Botash Botswana. Initial attempts to drill directly on the pan surface in 2015 and 2016 were unsuccessful because unseasonal rainfall prevented the establishment of a hard lake crust - a stable platform for the mobile drilling rig. To overcome these logistical constraints, two approaches were taken in July 2016 i) a series of short vibracores were taken with a lighter vehicle on the pan surface (data under preparation, Burrough et al., in prep) and ii), this study. Drilling using a mobile rig was undertaken through the sandy sediments of Sowa Spit, a cuspate spit formed during the Last Glacial Maximum^9^ allowing access to the underlying fine-grained lake sediments while providing a mechanically stable drilling surface. The overlying sands, down to 320 cm below surface (bls), were removed using four short 0.5 m cores and were subsampled for further OSL dating (eight OSL samples). The results presented in this study are from Core SUA16DS3 (320–720 cm below land surface, bls) which comprises two long overlapping cores measuring 1.93 m and 1.89 m, reaching a combined depth of 3.82 m into lake muds and 7.3 m from the surface.

Twelve samples for OSL dating were subsampled from these sediments. Cores were shipped to the UK and stored at the University of Oxford in cold conditions. Core splitting and sample preparation occurred at the Oxford Luminescence Dating Laboratory (OLD Lab). One half of each core was kept dark for OSL dating, the other half was analysed under normal light in a clean laboratory.

Although radiocarbon dating has been successfully applied elsewhere in the Makgadikgadi Basin, attempts to use ^14^C dating on the upper meter of the Sowa Spit core yielded indistinguishable ages. This likely reflected site-specific hydrological disturbance related to groundwater pumping and evaporative brine pools near the Botash mine. Radiocarbon dating was therefore not pursued further, and the chronology is based on OSL ages.

### OSL sample preparation

Following longitudinal core splitting in subdued red light (600 nm) conditions, 1 cm^3^ sub-samples were removed from the core sections for OSL dating. The location of samples was informed by the visible internal stratigraphy of the core. These sub-samples were then additionally split into components for dosimetry, sedimentology and Equivalent Dose (*D*_*e*_). The process of isolating quartz grains for D_e_ measurements involved saturating the bulk sediment subsample with 37% HCl and 30% H_2_O_2_ to eliminate carbonates and organics respectively. Following sieving with 90-micron mesh, coarse grains (> 90 μm) and fine grains (< 90 μm) were prepared separately. For the coarse separates, density separation with sodium polytungstate isolated the quartz component (density range: >2.62 and < 2.7 g cm^−^³) from feldspars and heavy minerals. The extracted quartz was then etched with 40% HF for 50 min to remove the α-irradiated outer rind and any residual feldspar within the subsample. Following this, a 24-hour HCl wash was applied to remove fluorides, and the sample was back-sieved using a 180 μm mesh. The remaining 180–212 μm quartz grains were then mounted into an array of 100 individual 300-µm holes on an aluminum disc for single-grain OSL analysis. The fine-grained (< 90 micron) isolate was shaken in Fluorosilicic acid (H_2_SiF_6_) for 10 days to remove feldspars. A final HCl wash neutralised any remaining fluorides. Multiple iterations of Stoke’s settling were then used to separate the 4–11 μm grains and a water-based solution of grains was used to create a monolayer of fine grain quartz (> 10,000 grains per aliquot) mounted on aluminium discs.

### Optically stimulated luminescence (OSL) measurements and equivalent dose (De) determination

OSL measurements were performed using a TL-DA-15 reader equipped with an EM19235QA photomultiplier and two 3-mm-thick U-340 filters. Laboratory beta irradiation was delivered via a calibrated ^90^Sr/^90^Y source.

Preheat conditions were optimized based on preheat plateau tests, which revealed very little systematic dependence on preheat temperature below 250 °C. Based on these, preheats of 240 °C for 10 s (PH1) and 200 °C for 0 s (PH2) were applied to single grain samples and 220 °C for 10 s (PH1) and 180 °C for 0 s (PH2) were applied to fine grained samples.

For fine-grained samples, arrays of blue light-emitting diodes (with a peak emission of 470 nm +/- 20 nm) were used for OSL signal stimulation which was delivered over 50 s. Data acquisition of the OSL signal spanned 1000 channels and the luminescence signals were integrated over the first five channels (0.25 s) to maximize fast-component isolation and reduce contamination from more slowly decaying medium components. Background noise was subtracted based on the final 500 channels (25 s) of the OSL decay curve.

For single grain measurements, made on 180–120 μm sand, a focused 532 nm laser was used for 1 s of stimulation with data recorded in 60 channels. Luminescence signals were integrated using the first five channels (0.1 s) and instrumental noise was measured using a background subtraction of the last 20 channels (0.4 s) of the OSL decay.

Sensitivity corrections were evaluated using recycling ratios, while potential feldspar contamination was assessed via IR-OSL depletion ratios^79^. Aliquots were rejected if the recycling or depletion ratios deviated by more than 10% from unity. Thermal transfer was quantified by measuring the OSL response to a zero-dose irradiation, expressed as a percentage of the natural signal. Aliquots were rejected if thermal transfer exceeded 5% of the natural signal. Additionally, grains were rejected if the test dose signal (Tn) was dim (if the initial Tn signal was less than 3σ above the corresponding background count). The application of these criteria typically led to the rejection of less than 10% of aliquots for multigrain measurements and 15–25% of grains for single grain measurements.

A dose recovery test (DRT) was conducted on five samples from the core, targeting the expected D_e_ value from initial range-finder tests. The selected protocol held up to these tests and the mean dose recovery ratio across samples was 0.97 ± 0.2.

#### Dose rate (D′) determination

The dose rate (D′) for OSL age determination was measured via Inductively Coupled Plasma Mass Spectrometry (ICP-MS) to quantify the concentrations of ^232^Th, ^238^U, and ^40^K within each sample, assuming a conservative relative error of 10% on these values because of micro-dosimetric variation. External alpha, beta and gamma dose rates were calculated using the conversion factors of^80^ and alpha and beta attenuation coefficients provided by^81^ and^82^ respectively. An alpha efficiency factor of 0.038 ± 0.02 was considered. We assumed secular equilibrium within the ^238^U and ^232^Th decay chains and assumed a 0.01 Gy ka^− 1^ internal dose rate for sand sized particles with a 20% relative uncertainty^8^.

The time-averaged water content for samples with a high silt and clay content was assumed to be 20% with a conservative relative uncertainty of 25%. For samples within the lake cores with more than 50% sand we assumed a 10% water content in line with as-found values. For samples in the overlying spit which contained much higher proportions of sand, and which are today above flood levels year-round, we assumed an average water content of 5%. The contribution of cosmic-ray dose was estimated as a function of depth, altitude and geomagnetic latitude using the formula proposed by^83^. The specific radionuclide activities and annual doses are presented in Table S1.

Single grain measurements on sand-sized material exhibited overdispersion values within the expected range (Table S1) for all but the uppermost sample (SUA16/Spit/94) which had a overdispersion value of 57% and showed some evidence of mixing within the stratigraphy, including significant root penetration and animal burrows. For this sample, we used the Finite Mixture Model (FMM) to extract multiple age components from the distribution. The optimum number of FMM components was selected taking into consideration the Bayesian Information Criterion and log likelihood statistical parameters. The *representative* component was then chosen taking into consideration the underlying stratigraphy and the proportion of grains within each FMM age group. For all other coarse grain samples, the Central Age Model (CAM) was used. Fine (4–11 μm) multigrain quartz dating averages the cumulative measurement of c.10,000 grains for each aliquot. The large number of grains per aliquot significantly improves the precision of the age estimate but the averaging effects can sometimes mask anomalous data that can inform us of inaccuracies within the dating assumptions (e.g. partial bleaching or biological mixing). However, as there was no obvious statistical or sedimentological reason to suspect either of these issues for samples in question, we used the central age model (CAM) to calculate sample ages for all fine-grained core samples.

A Bayesian age model for the entire section of OSL dates (including the overlying spit) was constructed in BACON (v.2.2) which produced best estimate modelled ages for each 1 cm section of the core^84^. Prior information provided to the model included specifying a hiatus at 369 cm (ca. 20 ka); an accretion shape of 1.5, an accumulation mean of 20 and 164 at the upper and lower side of the hiatus, memory strength of 7 and memory mean of 0.5. Relative uncertainties on the input OSL ages range between ~ 8 and 15%, consistent with typical OSL precision. When propagated through the Bayesian age model, these uncertainties result in modelled age ranges of ~ 1.5 kyr in the youngest sediments and up to ~ 9 kyr in the oldest part of the core.

### Geochemical and sedimentological analyses

The light exposed core length was subsequently sampled using plastic u-channels to provide a portable section with a flat uniform surface. High-resolution elemental concentration profiles were obtained from the U-channel sections using non-destructive X-ray fluorescence (XRF) spectrometry with an ITRAX core scanner (School of Environment, Education and Development, University of Manchester). X-ray irradiation was performed using a 3 kW Mo-tube, with data acquisition conducted at a step size of 1 mm and a count time of 20 s per step. Elemental concentrations were recorded and expressed as total counts per second (CPS). Measurement noise was removed if the mean standard error (MSE) of a data point was greater than five (higher MSE values may indicate issues such as overlapping peaks, matrix effects, or poor signal quality. The data was also filtered to remove datapoints if the validity measurement was less than 1 (lower values indicate potential distortions due to sample heterogeneity, water content, or misalignment during scanning). A loess smoother (local polynomial regression with a span of 0.003) was used prior to interpolation on to the Bayesian age model. The XRF counts obtained were processed following^85^, which means that the counts were log-ratio centered (clr) using the “clr” function of the Rpackage “compositions”^86^ including elements from Si to Pb. Principal component analysis (PCA with PC1: 35.4%, PC2: 15%) of log-ratio centered values of these geochemical parameters, reveals a positive correlation on PC1 between Ru, Cl, and loading opposite on PC1 between Zn, Cr, Cu, K, Ti, Rb, V (Fig. S2a). On PC2, Mn, Fe, Ni, S load opposite to Si, Zr and Ca, Sr, Ar. To develop XRF ratios, titanium (Ti) was used to normalise the XRF counts due to its conservative nature^41^. To account for non-linear matrix effects we used the log-ratio approach^85^.

A longitudinal portion of remaining core was sub-sectioned into 1 cm samples and particle size analysis (2–2000 μm grains) was carried out using a Malvern Laser Particle Size Analyser (Hydro 2000MU) at the University of Oxford, using 3 laser measurements on 3 independent subsamples from each 1 cm sample. Sediment statistics were calculated using the Folk and Ward formulae^87^.

Another PCA was processed (PC1: 36.2%, PC2: 24.2%, Fig. S2b) based on a combined dataset of grain size and the log-ratio centered values of each element. To do so, XRF data were down-sampled to the same depth resolution measured for grain size (*n* = 317).

### Organic geochemistry

#### Bulk organic carbon analysis

Carbon isotopic compositions (δ^13^C_bulk_, the ratio of ^13^C/^12^C in a sample relative to the standard Vienna PeeDee Belemnite, VPDB) and total organic carbon content (TOC) were measured using 50 to 100 mg of freeze-dried and homogenized sediment fumigated with concentrated hydrochloric acid (HCl 37%) for 72 h at 60 °C to remove inorganic carbon, and subsequently neutralized and dried under a basic atmosphere (pH > 7, NaOH) at 60 °C for another 72 h following^88^. Analysis were done with an Elemental Analyzer (EA) interface coupled to a stable isotope analyzer (EA-IRMS, Elementar vario MICRO cube—Isoprime Precision)^89^. The instrument accuracy during analysis was 0.13‰, the standards used were oxalic acid II (NIST SRM 4990 C), phthalic anhydride (Sigma, PN-320064–500 g, LN-MKBH1376V), atropine (Säntis, PN-SA990746B, LN-51112), and acetanilide (Merck, PN-100011, LN-K37102211229). The ratios were blank, size and drift corrected^88^.

39 freeze-dried and homogenized sediment samples (0.9 – 7 g) were extracted with an EDGE system (CEM) as described in^88^. Briefly, after extraction, total lipid extract was saponified with 0.5 M KOH in methanol (MeOH) and the neutral fraction was extracted with hexane. The remaining saponified products were acidified to pH 1 and fatty acids were extracted three times with hexane: dichloromethane (DCM) (4 : 1, *v/v*) and methylated overnight using MeOH of known isotopic composition. The resulting fatty acid methyl esters (FAMEs) were extracted four times with hexane^88^. FAME quantification using GC-FID has been described in^88^ using a known amount of C_36_ *n*-alkane (Sigma Aldrich) that was run multiple times as external standard during the same sequence^88^. The neutral fraction was separated into three fractions over activated silica oxide column (1% w), the most polar fraction was eluted with DCM : MeOH (1 : 1, *v/v*). Internal standards were added to the polar fraction: C_46_ GDGT^90^ and the C_22_ 5,16-diol^51^ (Interbioscreen). The polar fraction was then filtered using a polytetrafluoroethylene 0.45 μm filter prior to GDGT analysis on a normal phase high performance liquid chromatography (HPLC) following^91^. After GDGT analysis, the remaining polar fraction was silylated using BSTFA (30 min, 70 °C), and ran according to^92^ on a GC (Agilent 7890B) coupled to a mass spectrometer (MS, Agilent 5977B) equipped with a DB-5MS column (30 m x 0.25 mm, 0.25 μm film thickness) on both selected ion monitoring (SIM) and scan total ion chromatography (TIC) to quantify long-chain diols^51^ as well as sterols and hopanols, respectively. The SIM method was the same as in^51,92^, the Scan method frequency was 2 scans s^−1^ from *m/*z 50 to 800, with a gain factor of 3. The source was set at 230 °C and the Quadrupole at 150 °C.

### Climate model

HadCM3 is a GCM (General Circulation Model) consisting of coupled atmospheric, ocean and sea-ice model components^93,94^ with a coupled dynamic vegetation component^95^. The resolution of the atmospheric model is 2.5° in latitude by 3.75° in longitude by 19 unequally spaced vertical levels. The spatial resolution over the ocean in HadCM3 is 1.25° by 1.25° by 20 unequally spaced layers in the ocean extending to a depth of 5200 m. HadCM3^93,94^ was used coupled with the MOSES2.1 land surface scheme^96^ and the TRIFFID vegetation model^95^ which divide the land surface into nine surface types. The TRIFFID model was ‘dynamically’ coupled to the land surface. HadCM3 was forced with prescribed changes in orbital configuration, which alter the seasonal and latitudinal distribution of solar insolation. Also prescribed were variations in greenhouse gases (CO_2_, CH_4_, N_2_O), sea level, and ice-sheet extent and volume. See^97,98^ for further details of these boundary conditions.

A series of snapshot simulations were run (see also^99^) consisting of 62 model runs covering the last 120 kyr with the above multi-millennial scale forcings. All simulations were run to equilibrium for 500 years. To achieve Heinrich events and Younger Dryas simulations, 0.3 Sv freshwater ‘hosing’ was added to the North Atlantic region following the initial simulations at 13 (Younger Dryas), 17 (H1), 24 (H2), 32 (H3), 38 (H4), 46 (H5), and 60 (H6) kyr BP (using dates from^60^). We note the uncertainty in these dates, and that recently calibrated radiocarbon dates in one study provide earlier dates, especially for H4 at 43 kyr and H5 at 54 kyr^100^. The full set of simulations of the last glacial cycle have previously been demonstrated to compare well to spatial patterns and temporal trends in palaeo proxy data over Africa^99^ global tropical hydroclimates^101^, and at high latitudes^98^.

Average precipitation for the Angola Highlands and central Makgadikgadi regions were calculated on the original HadCM3 grid. In order to calculate total surface water balance (precipitation minus potential evapotranspiration; P-E) for the whole catchment shown in Fig. 1, the HadCM3 outputs were downscaled to 0.1° resolution using the Climate Data Operators^102^ software conservative interpolation (‘*remapcon’* function) to ensure that the area-weighted sum of values remained constant before and after interpolation. This allowed the extraction and summation of high spatial resolution hydroclimate outputs within the basin outline. The modelled modern surface water balance was found to be more positive than suggested by climate reanalysis (ERA5^103^), and consequently, a bias correction was applied to the downscaled HadCM3 catchment P-E. The 1979–2019 average for catchment P-E from ERA5 was added to the downscaled HadCM3 P-E anomaly for each palae-time slice from the modern pre-industrial HadCM3 P-E output.

## Supplementary Information

Below is the link to the electronic supplementary material.

Supplementary Material 1

## Data Availability

All data used in this study are available in the supplementary information.

## References

[1] Joyce, D. A. et al. An extant cichlid fish radiation emerged in an extinct Pleistocene lake. *Nature* **435**, 90–95 (2005). 15875022 10.1038/nature03489

[2] Robbins, L. H., Brook, G. A., Murphy, M. L., Ivester, A. H. & Campbell, A. C. The Kalahari During MIS 6 – 2 (190–12 ka): Archaeology, Paleoenvironment, and Population Dynamics. in Africa from MIS 6 – 2: Population Dynamics and Paleoenvironments (eds (eds Jones, S. C. & Stewart, B. A.) 175–193 (Springer Netherlands, Dordrecht, 10.1007/978-94-017-7520-5_10. (2016).

[3] Ackermann, R. et al. Upholding ‘good science’ in human origins research: A response to Chan et al. 10.31730/osf.io/qtjfp (2019).

[4] Thomas, D. S. G. et al. Lacustrine geoarchaeology in the central Kalahari: Implications for Middle Stone Age behaviour and adaptation in dryland conditions. *Quat. Sci. Rev.* **297**, 107826 (2022).

[5] Schlebusch, C. M. et al. Human origins in Southern African palaeo-wetlands? Strong claims from weak evidence. *J. Archaeol. Sci.* **130**, 105374 (2021).

[6] Chan, E. K. F. et al. Human origins in a Southern African palaeo-wetland and first migrations. *Nature* **575**, 185–189 (2019). 31659339 10.1038/s41586-019-1714-1

[7] Riedel, F. et al. Evidence for a permanent lake in Sua Pan (Kalahari, Botswana) during the early centuries of the last millennium indicated by distribution of baobab trees (*Adansonia digitata*) on “Kubu Island”. *Quat. Int.* **253**, 67–73 (2012).

[8] Burrough, S. L., Thomas, D. S. G. & Bailey, R. M. Mega-lake in the Kalahari: A Late Pleistocene record of the Palaeolake Makgadikgadi system. *Quat. Sci. Rev.* **28**, 1392–1411 (2009).

[9] Burrough, S. L. The Makgadikgadi Basin. in Landscapes and Landforms of Botswana (ed Eckardt, F. D.) 77–90 (Springer International Publishing, Cham, 10.1007/978-3-030-86102-5_5. (2022).

[10] Schmidt, M. et al. Paleolimnological features of a mega-lake phase in the Makgadikgadi Basin (Kalahari, Botswana) during Marine Isotope Stage 5 inferred from diatoms. *J. Paleolimnol.* **58**, 373–390 (2017).

[11] Thomas, D. & Shaw, P. A. *The Kalahari Environment* (Cambridge University Press, 1991).

[12] Riedel, F. et al. Dynamics of a Kalahari long-lived mega-lake system: hydromorphological and limnological changes in the Makgadikgadi Basin (Botswana) during the terminal 50 ka. *Hydrobiologia* **739**, 25–53 (2014).

[13] Grove, A. T. Landforms and climatic change in the Kalahari and Ngamiland. *Geogr. J.* **135**, 191–212 (1969).

[14] Cooke, H. J. & Verstappen, H. T. The landforms of the western Makgadikgadi Basin in northern Botswana, with a consideration of the chronology of the evolution of Lake Palaeo-Makgadikgadi. *Z. Geomorphol.* **28**, 1–19 (1984).

[15] McFarlane, M. J. & Eckardt, F. D. Lake Deception: A new Makgadikgadi Palaeolake. *Botsw. Notes Rec.* **38**, 195–201 (2006).

[16] Shaw, P. A. & Stokes, S. Paleoecology and age of a Quaternary high lake level in the Makgadikgadi Basin of the Middle Kalahari, Botswana. *South Afr. J. Sci.* **93**, 273–277 (1997).

[17] Du Toit, A. L. Crustal movements as a factor in the evolution of South Africa. *South Afr. Geogr. J.* **16**, 3–20.

[18] Schoville, B. J., Brown, K. S. & Wilkins, J. A lithic provisioning model as a proxy for landscape mobility in the Southern and Middle Kalahari. *J. Archaeol. Method Theory* **29**, 162–187 (2022).

[19] Staurset, S. et al. Making points: The Middle Stone Age lithic industry of the Makgadikgadi Basin, Botswana. *Quat. Sci. Rev.* **301**, 107823 (2023).

[20] Burrough, S. L. et al. Lessons from a lakebed: unpicking hydrological change and early human landscape use in the Makgadikgadi basin, Botswana. *Quat Sci. Rev.* **291**, 107662 (2022).

[21] Coulson, S. et al. Thriving in the Thirstland: New Stone Age sites from the Middle Kalahari, Botswana. *Quat. Sci. Rev.* **297**, 107695 (2022).

[22] Nash, D. J. et al. Mapping Middle Stone Age human mobility in the Makgadikgadi Pans (Botswana) through multi-site geochemical provenancing of silcrete artefacts. *Quat. Sci. Rev.* **297**, 107811 (2022).

[23] Burrough, S. L. Late Quaternary Environmental Change and Human Occupation of the Southern African Interior. in Africa from MIS 6 – 2 (eds (eds Jones, S. C. & Stewart, B. A.) 161–174 (Springer Netherlands, Dordrecht, 10.1007/978-94-017-7520-5_9. (2016).

[24] Jones, S. C. & Stewart, B. A. *Africa from MIS 6 – 2: Population Dynamics and Paleoenvironments* (Springer, 2016).

[25] Robbins, L. H. Stone Age Archaeology in the Northern Kalahari, Botswana: Savuti and Kudiakam Pan. *Curr. Anthropol.* **28**, 567–569 (1987).

[26] Gaudaré, L., Dauteuil, O. & Jolivet, M. Geomorphology of the Makgadikgadi Basin (Botswana): Insight into the propagation of the East African Rift System. *Tectonics* **43**, e2023TC007988 (2024).

[27] McFarlane, M. J. & Sedadika, P. Archaeological evidence for the reassessment of the ages of the Makgadikgadi Paleolakes. *Botsw. Notes Rec.* **33**, 83–90 (2001).

[28] Moore, A. E. & Larkin, P. A. Drainage evolution in South-central Africa since the breakup of Gondwana. *South. Afr. J. Geol.* **104**, 47–68 (2001).

[29] De Cort, G., Chevalier, M., Burrough, S. L., Chen, C. Y. & Harrison, S. P. An uncertainty-focused database approach to extract spatiotemporal trends from qualitative and discontinuous lake-status histories. *Quat Sci. Rev.* **258**, 106870 (2021).

[30] Burrough, S. L. & Thomas, D. S. G. Geomorphological contributions to palaeolimnology on the African continent. *Geomorphology* **103**, 285–298 (2009).

[31] Holmgren, K., Lauritzen, S. E. & Possnert, G. 230Th234U and 14 C dating of a late Pleistocene stalagmite in Lobatse II Cave, Botswana. *Quat Sci. Rev.* **13**, 111–119 (1994).

[32] Holmgren, K., Karlén, W. & Shaw, P. A. Paleoclimatic Significance of the Stable Isotopic Composition and Petrology of a Late Pleistocene Stalagmite from Botswana. *Quat Res.* **43**, 320–328 (1995).

[33] Wiese, R. et al. Lake highstands in the northern Kalahari, Botswana, during MIS 3b and LGM. *Quat Int.* **558**, 10–18 (2020).

[34] Brook, G. A. University of Georgia Paleoenvironmental Studies in Northwest Botswana 1987–1993. *Botsw. Notes Rec*. **24**, 233–235 (1992).

[35] Thomas, D. S. G. et al. Late Pleistocene wetting and drying in the NW Kalahari: an integrated study from the Tsodilo Hills, Botswana. *Quat Int.* **104**, 53–67 (2003).

[36] Shaw, P. A., Thomas, D. S. G. & Nash, D. J. Late Quaternary fluvial activity in the dry valleys (mekgacha) of the Middle and Southern Kalahari, southern Africa. *J. Quat Sci.* **7**, 273–281 (1992).

[37] Russell, J. M., Hopmans, E. C., Loomis, S. E., Liang, J. & Sinninghe Damsté, J. S. Distributions of 5- and 6-methyl branched glycerol dialkyl glycerol tetraethers (brGDGTs) in East African lake sediment: Effects of temperature, pH, and new lacustrine paleotemperature calibrations. *Org. Geochem.* **117**, 56–69 (2018).

[38] Collins, D. B. G. & Bras, R. L. Climatic control of sediment yield in dry lands following climate and land cover change. *Water Resour. Res.*10.1029/2007WR006474 (2008).

[39] Yuill, B. T. & Nichols, M. H. Patterns of grain-size dependent sediment transport in low-ordered, ephemeral channels. *Earth Surf. Process. Landf.* **36**, 334–346 (2011).

[40] Eglinton, T. I. & Eglinton, G. Molecular proxies for paleoclimatology. *Earth Planet. Sci. Lett.* **275**, 1–16 (2008).

[41] Kylander, M. E., Ampel, L., Wohlfarth, B. & Veres, D. High-resolution x-ray fluorescence core scanning analysis of Les Echets (France) sedimentary sequence: New insights from chemical proxies. *J. Quat. Sci.* **26**, 109–117 (2011).

[42] Evans, G., Augustinus, P., Gadd, P., Zawadzki, A. & Ditchfield, A. A multi-proxy µ-XRF inferred lake sediment record of environmental change spanning the last ca. 2230 years from Lake Kanono, Northland, New Zealand. *Quat. Sci. Rev.* **225**, 106000 (2019).

[43] Davison, W. Iron and manganese in lakes. *Earth-Sci. Rev.* **34**, 119–163 (1993).

[44] Rothwell, R. G. & Croudace, I. w. Twenty Years of XRF Core Scanning Marine Sediments: What Do Geochemical Proxies Tell Us? in Micro-XRF Studies of Sediment Cores: Applications of a non-destructive tool for the environmental sciences (eds (eds Croudace, I. W. & Rothwell, R. G.) 25–102 (Springer Netherlands, Dordrecht, 10.1007/978-94-017-9849-5_2. (2015).

[45] Ten Haven, H. L., Rohmer, M., Rullkötter, J. & Bisseret, P. Tetrahymanol, the most likely precursor of gammacerane, occurs ubiquitously in marine sediments. *Geochim. Cosmochim. Acta* **53**, 3073–3079 (1989).

[46] Rampen, S. W. et al. Sources and proxy potential of long chain alkyl diols in lacustrine environments. *Geochim. Cosmochim. Acta* **144**, 59–71 (2014).

[47] Lattaud, J. et al. Sources and seasonality of long-chain diols in a temperate lake (Lake Geneva). *Org. Geochem.* **156**, 104223 (2021).

[48] van Bree, L. G. J. et al. Seasonal variability in the abundance and stable carbon-isotopic composition of lipid biomarkers in suspended particulate matter from a stratified equatorial lake (Lake Chala, Kenya/Tanzania): Implications for the sedimentary record. *Quat. Sci. Rev.* **192**, 208–224 (2018).

[49] García-Alix, A. et al. Algal lipids reveal unprecedented warming rates in alpine areas of SW Europe during the industrial period. *Clim. Past* **16**, 245–263 (2020).

[50] Toney, J. L. et al. New insights into Holocene hydrology and temperature from lipid biomarkers in western Mediterranean alpine wetlands. *Quat. Sci. Rev.* **240**, (2020).

[51] Lattaud, J., Martin, C., Lloren, R., Zborovsky, B. & Dubois, N. Temperature and nutrients control the presence and distribution of long-chain diols in Swiss lakes. *Front. Earth Sci.* **12**, 1409137 (2024).

[52] Ficken, K. J., Li, B., Swain, D. L. & Eglinton, G. An n-alkane proxy for the sedimentary input of submerged/floating freshwater aquatic macrophytes. *Org. Geochem.* **31**, 745–749 (2000).

[53] Schouten, S., Hopmans, E. C., Damsté, J. S. S. & Sinninghe Damsté, J. S. The organic geochemistry of glycerol dialkyl glycerol tetraether lipids: A review. *Org. Geochem.* **54**, 19–61 (2013).

[54] Ding, S. et al. Identification of novel 7-methyl and cyclopentanyl branched glycerol dialkyl glycerol tetraethers in lake sediments. *Org. Geochem.* **102**, 52–58 (2016).

[55] Wang, H. et al. Salinity-controlled isomerization of lacustrine brGDGTs impacts the associated MBT5ME’ terrestrial temperature index. *Geochim. Cosmochim. Acta*. **305**, 33–48 (2021).

[56] Moses, O. Projected changes in rainfall and temperature using CMIP6 models over the Okavango River basin, southern Africa. *Theor. Appl. Climatol*. **155**, 5337–5351 (2024).

[57] McCulloch, G. P., Irvine, K., Eckardt, F. D. & Bryant, R. Hydrochemical fluctuations and crustacean community composition in an ephemeral saline lake (Sua Pan, Makgadikgadi Botswana). *Hydrobiologia* **596**, 31–46 (2008).

[58] Thomas, A. D., Dougill, A. J., Elliott, D. R. & Mairs, H. Seasonal differences in soil CO2 efflux and carbon storage in Ntwetwe Pan, Makgadikgadi Basin, Botswana. *Geoderma* **219–220**, 72–81 (2014).

[59] West, D., van As, J. & van As, L. Surface water quality in the Okavango Delta Panhandle, Botswana. *Afr. J. Aquat. Sci.* **40**, 359–372 (2015).

[60] Hemming, S. R. Heinrich events: Massive late Pleistocene detritus layers of the North Atlantic and their global climate imprint. *Rev. Geophys.*10.1029/2003RG000128 (2004).

[61] Lattaud, J., Gondwe, M. J., Saurer, M., Helfter, C. & De Jonge, C. The preservation of photosynthetic and hydrological signals in the carbon and hydrogen isotope compositions of n-fatty acids in the seasonal wetland soils of the Okavango Delta (Botswana). *Org. Geochem.* 104832. 10.1016/j.orggeochem.2024.104832 (2024).

[62] Gälman, V., Rydberg, J. & Bigler, C. Decadal diagenetic effects on δ13C and δ15N studied in varved lake sediment. *Limnol. Oceanogr.* **54**, 917–924 (2009).

[63] Yoshioka, T. Phytoplanktonic carbon isotope fractionation: Equations accounting for CO2-concentrating mechanisms. *J. Plankton Res.* **19**, 1455–1476 (1997).

[64] Laskar, J. et al. A long-term numerical solution for the insolation quantities of the Earth. *Astron. Astrophys.* **428**, 261–285 (2004).

[65] Ringrose, S. Landscape Evolution of the Lake Ngami and Mababe Depressions Within the Okavango Rift Zone, North-Central Botswana. in Landscapes and Landforms of Botswana (ed Eckardt, F. D.) 57–75 (Springer International Publishing, Cham, 10.1007/978-3-030-86102-5_4. (2022).

[66] Boni, M., Bouabdellah, M., Boukirou, W., Putzolu, F. & Mondillo, N. Vanadium ore resources of the African continent: State of the art. *Ore Geol. Rev.* **157**, 105423 (2023).

[67] Garzanti, E. et al. The segmented Zambezi Sedimentary System from source to sink: 2. Geochemistry, clay minerals, and detrital geochronology. *J. Geol.* **130**, 171–208 (2022).

[68] Humphries, M. S. et al. Dust provenance and its role as a potential fertilizing agent for the Okavango Delta, Botswana. *Earth Surf. Process. Landf.* **45**, 1705–1716 (2020).

[69] Garzanti, E., Padoan, M., Setti, M., López-Galindo, A. & Villa, I. M. Provenance versus weathering control on the composition of tropical river mud (southern Africa). *Chem. Geol.* **366**, 61–74 (2014).

[70] Gao, Y. et al. Provenance analysis of late Pleistocene sediment from IODP site U1477 reveals climate and river basin dynamics in the Zambezi River catchment. *Gondwana Res.* **146**, 25–38 (2025).

[71] Helgren, D. M. Historical geomorphology and geoarchaeology in the Southwestern Makgadikgadi Basin, Botswana. *Ann. Assoc. Am. Geogr.* **74**, 298–307 (1984).

[72] Van Waarden, C. Stone Age people at Makalamabedi Drift. *Botsw. Notes Rec.* **23**, 251–4 (1991).

[73] COOKE, C. K. A preliminary report on the Stone Age of the Nata River, Botswana. *Prelim. Rep. Stone Age Nata River Botsw.* 2, 2 pl (1967).

[74] Main, M. Archaeological sites located in South Sowa Pan between 1993 and 1996: A summary. *Botsw. Notes Rec.* **40**, 46–54 (2008).

[75] Walker, N. Game traps: Their importance in Southern Africa. *Bostwana Notes Rec.* **23**, 235–242 (1991).

[76] Vickery, K. J. & Eckardt, F. D. Dust emission controls on the lower Kuiseb River valley, Central Namib. *Aeolian Res.* **10**, 125–133 (2013).

[77] Uda, T. *Morphodynamic Model for Predicting Beach Changes Based on Bagnold’s Concept and Its Applications* (BoD – Books on Demand, 2018).

[78] Zenkovich, V., Pavlovich, V., King, C. A. M. & Steers, J. *Processes of Coastal Development* (Oliver and Boyd, 1967).

[79] Duller, G. A. T. Distinguishing quartz and feldspar in single grain luminescence measurements. *Radiat. Meas.* **37**, 161–165 (2003).

[80] Liritzis, I. et al. Dose Rate. In *Luminescence Dating in Archaeology, Anthropology, and Geoarchaeology: An Overview* (eds Liritzis, I. et al.) 21–24 (Springer International Publishing, 2013). 10.1007/978-3-319-00170-8_3.

[81] Brennan, B. J., Lyons, R. G. & Phillips, S. W. Attenuation of alpha particle track dose for spherical grains. *Int. J. Radiat. Appl. Instrum. Part D Nucl. Tracks Radiat. Meas.* **18**, 249–253 (1991).

[82] Guérin, G., Mercier, N., Nathan, R., Adamiec, G. & Lefrais, Y. On the use of the infinite matrix assumption and associated concepts: A critical review. *Radiat. Meas.* **47**, 778–785 (2012).

[83] Prescott, J. R. & Hutton, J. T. Cosmic ray contributions to dose rates for luminescence and ESR dating: Large depths and long-term time variations. *Radiat. Meas.* **23**, 497–500 (1994).

[84] Blaauw, M. & Christen, J. A. Flexible paleoclimate age-depth models using an autoregressive gamma process. *Bayesian Anal.* **6**, 457–474 (2011).

[85] Bertrand, S. et al. Inorganic geochemistry of lake sediments: A review of analytical techniques and guidelines for data interpretation. *Earth-Sci. Rev.* **249**, 104639 (2024).

[86] Gerald, K. & van den Boogaart Raimon Tolosana-Delgado, Matevz Bren. compositions: Compositional Data Analysis. 2.0–8. 10.32614/CRAN.package.compositions (2005).

[87] Folk, R. L. & Ward, W. C. Brazos River bar [Texas]; a study in the significance of grain size parameters. *J. Sediment. Res.* **27**, 3–26 (1957).

[88] Lattaud, J. et al. Influence of hydraulic connectivity on carbon burial efficiency in Mackenzie Delta Lake Sediments. *J. Geophys. Res. Biogeosci.* **126**, (2021).

[89] McIntyre, C. et al. Online 13C and 14C gas measurements by EA-IRMS–AMS at ETH Zürich. *Radiocarbon* **59**, 893–903 (2016).

[90] Huguet, C. et al. An improved method to determine the absolute abundance of glycerol dibiphytanyl glycerol tetraether lipids. *Org. Geochem.* **37**, 1036–1041 (2006).

[91] Hopmans, E. C., Schouten, S. & Sinninghe Damsté, J. S. The effect of improved chromatography on GDGT-based palaeoproxies. *Org. Geochem.* **93**, 1–6 (2016).

[92] Rampen, S. W. et al. Long chain 1,13- and 1,15-diols as a potential proxy for palaeotemperature reconstruction. *Geochim. Cosmochim. Acta* **84**, 204–216 (2012).

[93] Gordon, C. et al. The simulation of SST, sea ice extents and ocean heat transports in a version of the Hadley Centre coupled model without flux adjustments. *Clim. Dyn.* **16**, 147–168 (2000).

[94] Pope, V. D., Gallani, M. L., Rowntree, P. R. & Stratton, R. A. The impact of new physical parametrizations in the Hadley Centre climate model: HadAM3. *Clim. Dyn.* **16**, 123–146 (2000).

[95] Cox, P. M. *Description of the TRIFFID Dynamic Global Vegetation Model*. (2001).

[96] Essery, R., Best, M. & Cox, P. M. *MOSES 2.2 Technical Documentation*. (2001).

[97] Eriksson, A. et al. Late Pleistocene climate change and the global expansion of anatomically modern humans. *Proc. Natl. Acad. Sci. U. S. A.* **109**, 16089–16094 (2012). 22988099 10.1073/pnas.1209494109 PMC3479575

[98] Singarayer, J. S. & Valdes, P. J. High-latitude climate sensitivity to ice-sheet forcing over the last 120 kyr. *Quat. Sci. Rev.* **29**, 43–55 (2010).

[99] Singarayer, J. S. & Burrough, S. L. Interhemispheric dynamics of the African rainbelt during the late Quaternary. *Quat. Sci. Rev.* **124**, 48–67 (2015).

[100] Sayago-Gil, M., López-González, N., Long, D., Fernández-Salas, L. M. & Durán-Muñoz, P. Multi-proxy approach for identifying Heinrich events in sediment cores from Hatton Bank (NE Atlantic Ocean). *Geosciences* **10**, 14 (2020).

[101] Singarayer, J. S., Valdes, P. J. & Roberts, W. H. G. Ocean dominated expansion and contraction of the late Quaternary tropical rainbelt. *Sci. Rep.* **7**, 9382 (2017). 28839263 10.1038/s41598-017-09816-8 PMC5571209

[102] Schulzweida, U. CDO User Guide. 10.5281/ZENODO.10020800 (2023).

[103] C3S. ERA5 hourly data on single levels from 1940 to present. Copernicus Climate Change Service (C3S) Climate Data Store (CDS). 10.24381/CDS.ADBB2D47 (2018).

[104] ArcGIS Pro. (2025).

[105] Halamka et al. Oxygen limitation can trigger the production of branched GDGTs in culture. *Geochem. Perspect. Lett.* **19**, (2021).

[106] Lattaud, J., Gondwe, M. J., Griepentrog, M., Helfter, C. & De Jonge, C. Soil chemistry effect on GDGT abundances and their proxies in soils of the Okavango Delta. *Org. Geochem.* **195**, 104847 (2024).

[107] Lattaud, J. et al. Long-chain diols in rivers: Distribution and potential biological sources. *Biogeosciences* **15**, 4147–4161 (2018).

[108] Volkman, J. K., Barrett, S. M., Dunstan, G. A. & Jeffrey, S. W. C30-C32 alkyl diols and unsaturated alcohols in microalgae of the class Eustigmatophyceae. *Org. Geochem.* **18**, 131–138 (1992).

[109] Volkman, J. K., Barrett, S. M. & Blackburn, S. I. Eustigmatophyte microalgae are potential sources of C29 sterols, C22-C28 n-alcohols and C28-C32 n-alkyl diols in freshwater environments. *Org. Geochem.* **30**, 307–318 (1999).

[110] Volkman, J. K. A review of sterol markers for marine and terrigenous organic matter. *Org. Geochem.* **9**, 83–99 (1986).

[111] Bull, I. D., Lockheart, M. J., Elhmmali, M. M., Roberts, D. J. & Evershed, R. P. The origin of faeces by means of biomarker detection. *Environ. Int.* **27**, 647–654 (2002). 11934114 10.1016/s0160-4120(01)00124-6

[112] Kemp, A. C. et al. Fecal steroids as a potential tool for conservation paleobiology in East Africa. *Biodivers. Conserv.* **31**, 183–209 (2022).

[113] Banta, A. B., Wei, J. H. & Welander, P. V. A distinct pathway for tetrahymanol synthesis in bacteria. *Proc. Natl. Acad. Sci. U. S. A.* **112**, 13478–13483 (2015). 26483502 10.1073/pnas.1511482112 PMC4640766

[114] Sinninghe Damsté, J. S. et al. Evidence for gammacerane as an indicator of water column stratification. *Geochim. Cosmochim. Acta* **59**, 1895–1900 (1995). 11540109 10.1016/0016-7037(95)00073-9

