# Supplementary material for: A multiproxy sediment-core record of lake-level change in paleolake Makgadikgadi (82–21 ka) with implications for human occupation
Source: Sci Rep. 2026 May 18;16:22366. doi: 10.1038/s41598-026-50559-2 (PMC13377114; doi:10.1038/s41598-026-50559-2)
Supplement: Supplementary file 1 — Supplementary Material 1 [file 41598_2026_50559_MOESM1_ESM.pdf]

Supporting Information for
A multiproxy sediment-core record of lake-level change in paleolake Makgadikgadi (82–21 ka) with implications for human occupation

‘

Julie Lattaud[1,2], Sallie Burrough[3], Ella Walsh[3,4], Josh Allin[3], Joy S. Singarayer[5], David S.G. Thomas[3,6], Negar Haghipour[1,7], Moruti Ntloedibe[8,] Godfrey Nkala[9], Chris Mpelege[8], Cindy De Jonge[1].

This PDF file includes:

Figures S1 to S3
Supplementary Results

Other supporting materials for this manuscript include the following:
Datasets Table S1 to S3
References

Supplementary results

Core description

SUA16DS3 comprises two long overlapping subcores measuring 1.93 m (PC1) and 1.89 m (PC2), reaching a combined depth of 3.82 m (Fig. S1a).

Unit I (0-50 cm): The upper 2 cm of the core are composed of oxidized sand. From 2 to 30 cm, the sediment is light grey sand without any visible internal structure (Fig. S1). This unit is characterized by elevated log(Zr/Rb) and log(Ca/Ti) values. The colored material visible in unit I corresponds to plastic stabilization inserts used during coring of unconsolidated sediments and does not represent a sedimentary or evaporitic layer. From 30 to 50 cm, the core is darker brown with white silt, and no internal structure is observed.

Unit II (50-210 cm): This unit is characterized by an alternation of brown to light brown silt with red layers at 65-70 cm, 97-102 cm, and 137-142 cm. The XRF ratios remain stable throughout this unit. From 147 to 210 cm, the sediment is grey-brown silt with a red layer at 185-190 cm. In this interval, the log(K/Ru) values decrease, while the log(Ca/Ti) and log(Cl/Ti) values increase.

Unit III (210 -250 cm): This unit is composed of brown clay with a high clay fraction, low log(Ca/Ti), log(Cl/Ti), and log(Zr/Rb) values, and elevated log(K/Ru).

Unit IV (250-350 cm) This unit consists of brown to light brown silty clay with a red layer from 287 to 350 cm. The sediment in this unit is characterized by elevated log(Ca/Ti), log(Cl/Ti), and log(Zr/Rb) values, and low log(K/Ru).

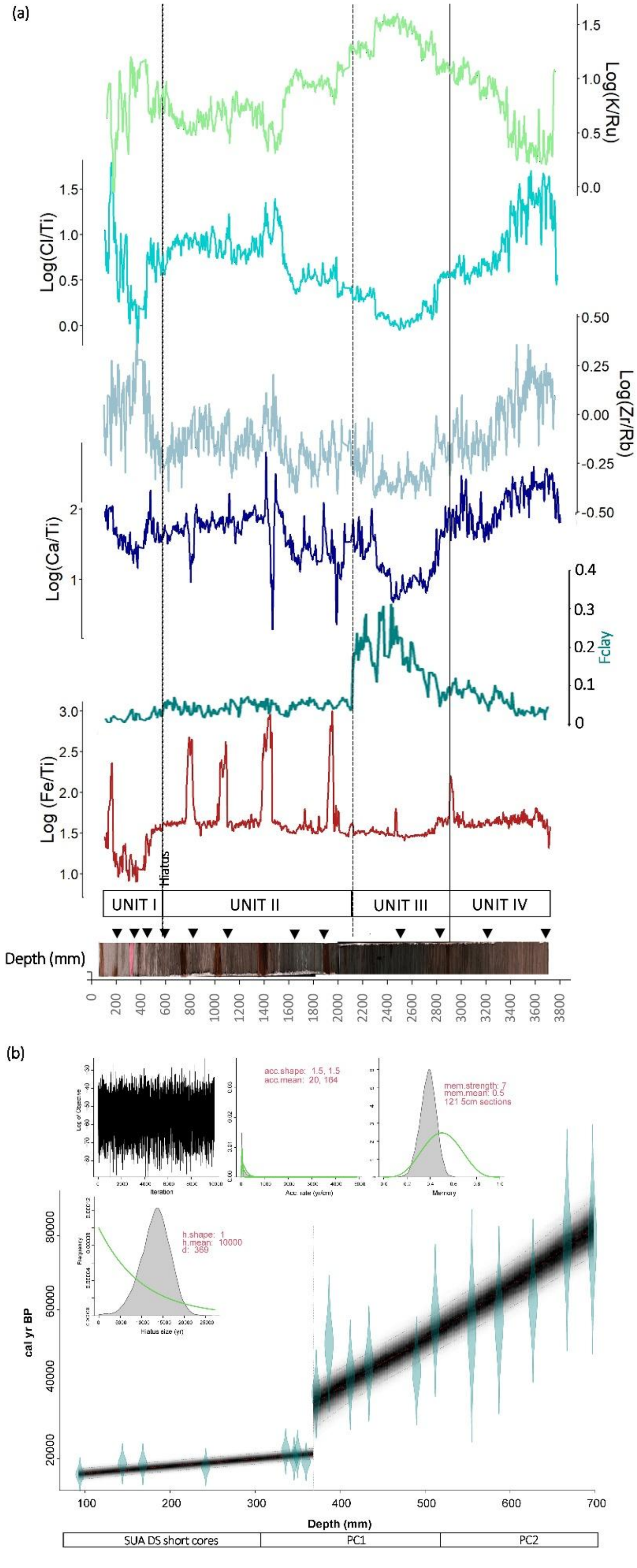
(a)
Log(K/Ru)
Log(Cl/Ti)
Log(Zr/Rb)
Log(Ca/Ti)
Fclay
Log (Fe/Ti)
Hiatus
UNIT I
UNIT II
UNIT III
UNIT IV
Depth (mm)
(b)
acc.shape: 1.5, 1.5
acc.mean: 20, 164
mem.strength: 7
mem.mean: 0.5
121 5cm sections
h.shape: 1
h.mean: 10000
d: 369
Hiatus size (yr)
cal yr BP
Depth (mm)
SUA DS short cores
PC1
PC2

Fig. S1. (a) High resolution core image and sedimentary unit depth definition using log (K/Ru), log(Cl/Ti), log(Zr/Rb), log(Ca/Ti), relative abundance of clay (Fclay) and log(Fe/Ti). The black triangle indicates the OSL dates, the red layer in the core image is a red plastic inserted to keep the structure of the core as the upper part was unconsolidated, (b) Bayesian Age model of core SUA16DS3 constructed using BACON v.2.2 [1]. Prior information provided to the model included specifying a hiatus at 369 cm below land surface, bls (ca.20 ka); an accretion shape of 1.5, an accumulation mean of 20 and 164 at the upper and lower side of the hiatus, memory strength of 0.1 and memory mean of 0.5. Blue shapes show the measured OSL ages and associated uncertainties. Depth is given below lake floor (bsl). The upper 8 dates originate from the short SUA DS cores taken above the longer SUA16DS3 core.

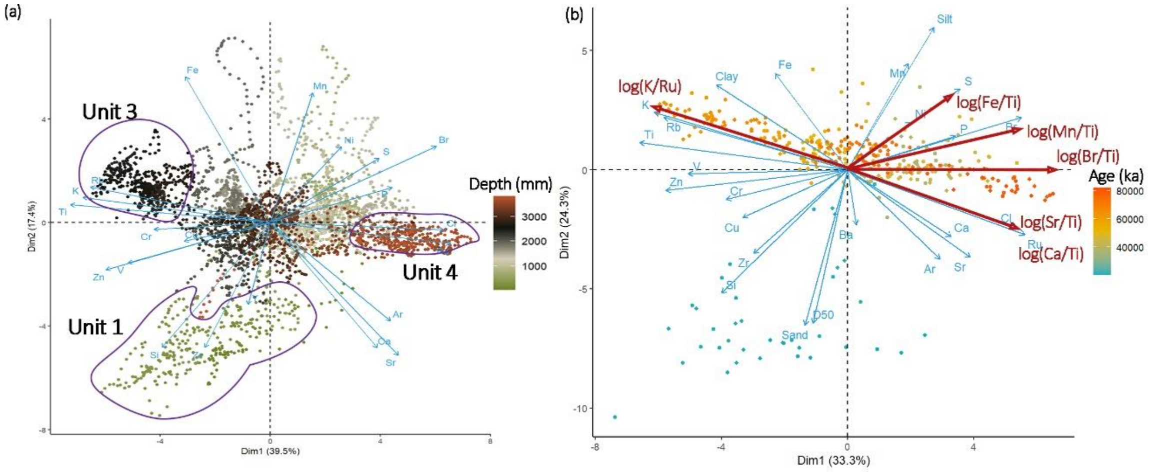

Fig. S2. (a) PCA of all log-ratio centered XRF counts and (b) PCA of log-ratio centered XRF counts (downsized to fit the number of analysis for grain size), XRF log ratios (Ti normalized) and grain size

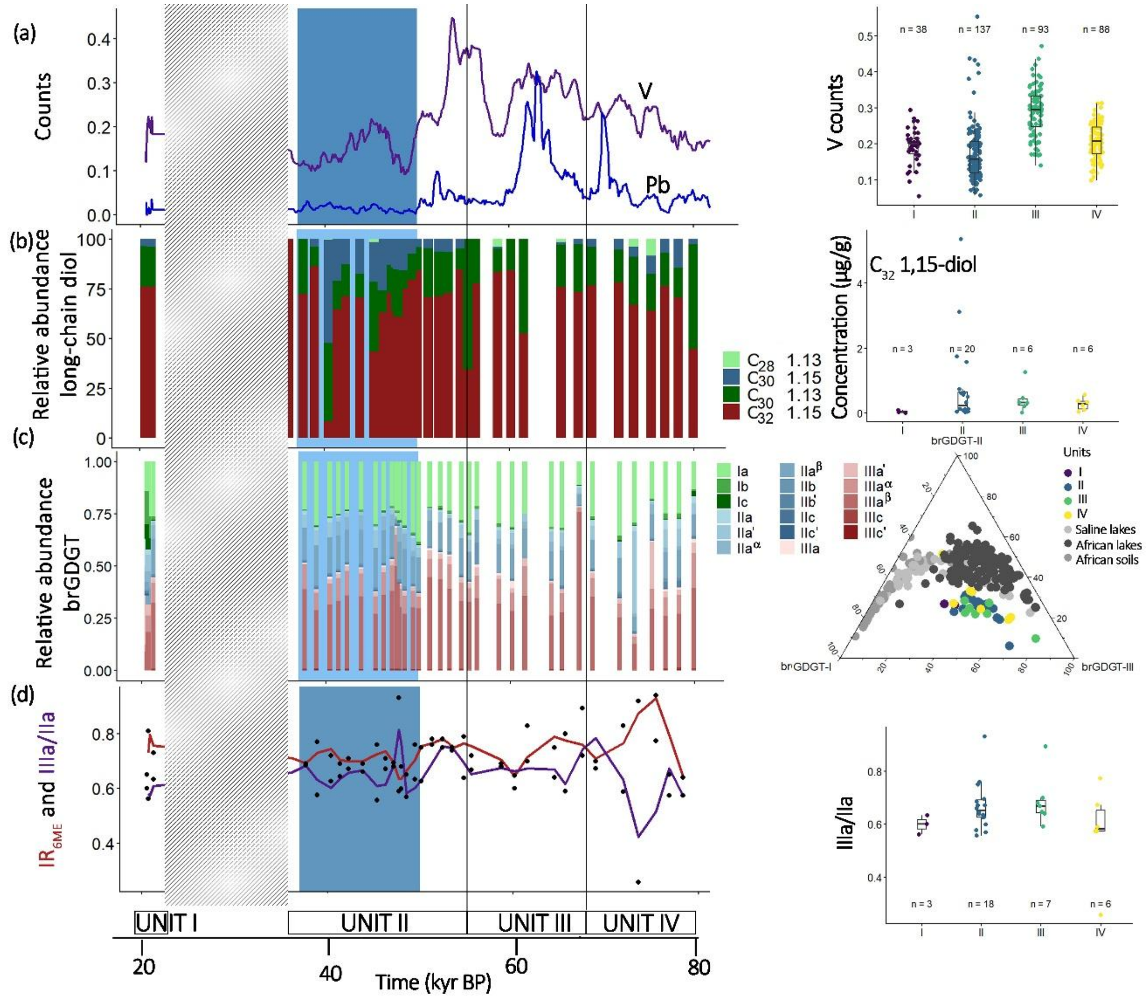

Figure S3: (a) XRF count of V and Pb downcore distribution (2-points average) and boxplot representing the average and distribution in the sedimentary units of V counts, (b) Relative abundance of the long-chain diol isomers downcore and boxplot representing the average and distribution in the sedimentary units of the proportion of C_{32} 1,15-diol, (c) Relative abundance of the brGDGTs downcore and associated ternary plot (African lake sediments from [2,3], African soil from [4–6]and saline lakes from [7–11]). Colors represent brGDGT subtypes, grouped by major structural class (I, II, III) using hue-consistent gradients and (d) isomer ratio (IR_{6ME}), IIa/(IIa+IIIa) ratio[12] downcore (2-points average) and boxplot representing the average and distribution in the sedimentary units of the IIa/(IIa+IIIa) ratio. The striped area indicates the sedimentary hiatus and the blue shaded area the potential anoxia period.

Table S1. Detailed results from optically stimulated luminescence (OSL) analysis

Sample Code	OSL Measurement type*	Age (ka)	Uncertainty (ka)	Depth from surface (cm)	n aliquots/grains	D_e (Gy)	Uncertainty (Gy)	Overdispersion (%)	% K	Th (ppm)	U (ppm)	Cosmic dose rate (mGy/ka)	error	Moisture content (%)	Total dose rate (Gy/ka)	Uncertainty (Gy/ka)	% error
SUA16/Spit/94	SG/SAR/FMM	15.7	1.3	94	54	25.5	0.9	57	1.08	3.10	0.87	0.215	0.020	5	1.62	0.12	7.48
SUA/Spit/148	SG/SAR/CAM	18.6	1.5	148	59	25.6	0.9	19	0.93	2.32	0.66	0.202	0.017	5	1.38	0.10	7.57
SUA/Spit/168	SG/SAR/CAM	18.5	1.6	168	67	25.0	1.0	26	0.92	2.28	0.64	0.196	0.016	5	1.35	0.10	7.59
SUA/Spit/242	SG/SAR/CAM	17.7	1.4	242	65	23.3	0.8	19	0.84	2.69	0.75	0.179	0.014	5	1.31	0.10	7.26
SUA/Spit/336	SG/SAR/CAM	21.9	1.7	336	64	27.9	0.9	16	0.82	2.11	0.90	0.160	0.012	5	1.27	0.09	7.32
SUA/Spit/350	SG/SAR/CAM	19.9	1.6	346	11	32.4	1.1	3	0.77	2.60	1.74	0.156	0.014	10	1.63	0.12	7.40
SUA16/Lake/PC1/010	MGF/SAR/CAM	21.3	1.8	350	67	28.4	1.0	21	0.94	2.25	1.02	0.158	0.012	10	1.33	0.10	7.76
SUA16/Lake/PC1/012	MGF/SAR/CAM	19.1	1.5	360	15	34.1	0.8	0	0.98	3.15	1.48	0.153	0.014	10	1.78	0.13	7.22
SUA16/Lake/PC1/015	MGF/SAR/CAM	37.4	3.4	372	25	106.0	2.4	0	1.25	11.00	3.19	0.151	0.014	20	2.83	0.25	8.78
SUA16/Lake/PC1/020	MGF/SAR/CAM	51.0	5.2	387	21	93.2	2.2	3	0.75	2.58	3.34	0.148	0.013	20	1.83	0.18	9.93
SUA16/Lake/PC1/025	MGF/SAR/CAM	42.6	4.2	412	17	93.9	2.5	4	1.19	3.08	3.32	0.144	0.013	20	2.21	0.21	9.47
SUA16/Lake/PC1/03	MGF/SAR/CAM	45.6	4.4	434	14	87.7	2.6	0	1.06	2.85	2.72	0.139	0.013	20	1.93	0.18	9.27
SUA16/Lake/PC1/04	MGF/SAR/CAM	43.6	4.2	490	18	118.1	3.0	10	1.59	5.28	3.42	0.130	0.012	20	2.71	0.25	9.19
SUA16/Lake/PC1/05	MGF/SAR/CAM	57.2	5.8	512	21	145.2	4.7	11	1.56	5.69	2.77	0.127	0.012	20	2.54	0.23	8.98
SUA16/Lake/PC2/01	MGF/SAR/CAM	55.8	8.9	555	24	295.9	4.6	0	2.64	10.13	8.74	0.121	0.011	20	5.31	0.81	15.30
SUA16/Lake/PC2/02	MGF/SAR/CAM	57.3	7.2	587	13	210.7	6.4	0	2.45	7.07	4.01	0.116	0.011	20	3.68	0.45	12.19
SUA16/Lake/PC2/03	MGF/SAR/CAM	62.4	6.3	627	15	179.7	5.1	0	1.38	6.19	4.53	0.111	0.011	20	2.88	0.28	9.63

SUA16/Lake/PC2/04	MGF/SAR/CAM	78.7	8.5	667	18	185.3	4.9	13	0.77	5.20	4.69	0.106	0.010	20	2.35	0.25	10.45
SUA16/Lake/PC2/05	MGF/SAR/CAM	79.0	8.8	697	12	118.7	3.8	12	0.47	2.52	3.16	0.103	0.010	20	1.50	0.16	10.64

*SG = Single Grain 180-210 µm; MGFG = Multigrain fines 4-11 µm; SAR = Single Aliquot Regeneration; CAM = Central Age Model; FMM = Finite Mixture Model

Table S2 Bulk organic parameters

Core	Depth (mm)	Median age (yr BP)	error age (yr BP)	$\delta^{13}C$ (‰)	TOC (%)
PC1 upper	50	20526	720	-19.64	0.06
PC1 upper	130	20686	1469		1.88
PC1 upper	160	20743	1452	-21.1	0.08
PC1 upper	200	20821	1465		0.38
PC1 upper	225	20868	1469	-20.91	
PC1 upper	255	20926	1461	-21.86	0.1
PC1 upper	390	21198	1462	-22.51	0.18
PC1 upper	410	21240	1455	-20.58	0.34
PC1 upper	480	35249	6240		
PC1 upper	520	35840	6032	-22.33	1.69
PC1 upper	540	36152	5977	-21.2	0.97
PC1 upper	640	37662	5798	-20.38	1.2
PC1 upper	710	38623	5627	-21.9	0.71
PC1 upper	730	38902	5588		1.21
PC1 upper	840	40395	5361	-20.94	1.32
PC1 upper	890	41086	5297	-21.93	0.48
PC1 upper	910	41346	5296	-18.5	1.01
PC1 upper	980	42270	5258	-20.71	0.62
PC1 upper	1100	43802	5187	-20.63	0.58
PC1 upper	1220	45342	5220	-20.7	0.81
PC1 upper	1290	46240	5230	-20.5	
PC1 upper	1358	47137	5251	-20.99	0.66
PC1 upper	1404	47705	5343	-20.2	0.78
PC1 upper	1422	47952	5199	-20.58	0.84
PC1 upper	1466	48501	5258	-21.02	1.21
PC1 upper	1540	49428	5309	-18.5	0.75
PC1 upper	1591	50067	5318	-18.05	0.4
PC1 upper	1680	51245	5376	-18.8	0.35
PC1 upper	1761	52386	5398	-18.45	0.31
PC1 lower	1801	53107	5381	-18.56	0.3
PC1 lower	1821	53397	5420	-20.34	0.17
PC1 lower	1921	54671	5506	-18.84	
PC1 lower	1971	55515	5532	-19.23	0.35
PC2	2010	56363	5527	-18.5	
PC2	2100	57343	5625	-18.61	0.2
PC2	2200	58740	5727	-18.41	0.22
PC2	2300	60163	5766	-19.32	0.29
PC2	2400	61559	5891	-19.06	0.34

PC2	2500	62989	5963	-18.8	0.25
PC2	2600	64427	6058	-20.98	0.26
PC2	2680	65610	6188	-20.43	0.38
PC2	2800	67466	6273		0.42
PC2	2910	68905	6448	-20.48	
PC2	3000	70264	6541	-21.02	0.75
PC2	3100	71862	6753	-20.9	0.81
PC2	3200	73472	6864	-21.17	0.64
PC2	3310	75382	7031	-22.29	0.59
PC2	3410	76835	7128	-22.67	0.75
PC2	3500	78300	7290	-22.7	0.89
PC2	3600	79896	7471	-22.84	0.93
PC2	3700	81493	7615	-23.46	0.93

Table S3 Biomarker concentration and proxies

		Concentration (μg/g)												
Median age (yr BP)	error age (yr BP)	24-ethyl-coprostanol	Tetrahymanol	Terrestrial sterols	Long chain fatty acids	C_{32} 1,15	C_{30} 1,13	C_{30} 1,15	C_{28} 1,13	Sum diols	Paq	IR'6,7 ME	Reconstructed pH	Reconstructed MAAT (°C)
20526	720	0.01	0.14	0.10	0.23	0.03	0.01	0.00	0.00	0.04	0.53	0.55	9.2	22.2
20686	1469	0.00	0.00	0.00	0.11	0.00	0.00	0.00	0.00	0.00	0.67	0.83	8.3	28.9
20868	1469				1.44	0.00	0.00	0.00	0.00	0.00	0.60			
21240	1455	0.74	1.08	2.50	3.71	0.09	0.02	0.00	0.00	0.12	0.37	0.94	7.2	29.1
36152	5977	0.00	1.26	0.00	4.41	0.03	0.00	0.00	0.00	0.03	0.40			
37662	5798	3.03	15.15	7.20	0.00	0.73	0.28	0.00	0.00	1.01				26.9
38902	5588	2.26	8.61	8.50	5.31	3.12	0.36	0.14	0.00	3.62	0.49	0.90	7.6	28.1
40395	5361	1.81	8.65	5.60	6.51	0.13	0.60	0.80	0.00	1.54	0.37	0.88	7.7	26.8
41346	5296	4.12	7.83	13.10	7.14	0.49	0.11	0.16	0.00	0.76	0.30	0.87	7.8	28.4
42270	5258	2.52	4.02	8.80	4.97	0.56	0.12	0.10	0.00	0.78	0.23	0.93	7.1	28.1
43802	5187	0.53	1.84	2.10	4.16	0.12	0.02	0.03	0.00	0.16	0.30	0.93	7.3	28.3
45342	5220	3.69	8.99	8.60	5.68	1.57	1.28	0.72	0.05	3.62	0.37	0.94	7.2	27.6
46240	5230	0.54	1.55	2.10	12.14	0.04	0.01	0.02	0.00	0.06	0.33	0.86	7.8	28.2
47137	5251	0.44	0.59	1.20	2.60	0.12	0.02	0.02	0.00	0.16	0.36	0.93	7.3	27.6
47705	5343	1.27	2.79	3.60	1.15	0.08	0.03	0.02	0.00	0.12	0.67	0.93	7.4	27.1
47952	5199	2.64	9.15	8.30	5.31	0.62	0.24	0.16	0.00	1.02	0.38	0.92	7.1	28.6
48501	5258	16.95	16.64	36.10	6.05	1.74	0.21	0.36	0.00	2.32	0.35	0.93	7.0	28.6
49428	5309	0.12	0.48	0.50	1.60	0.07	0.01	0.01	0.00	0.09	0.55	0.92	6.9	28.7
50067	5318	0.27	0.38	1.60	2.53	0.61	0.08	0.03	0.00	0.72	0.45	0.88	7.4	29.3
51245	5376	0.06	0.55	0.20	3.21	0.04	0.02	0.00	0.00	0.06	0.49	0.90	7.1	29.2
52386	5398	0.09	0.63	0.40	2.14	0.04	0.01	0.00	0.00	0.06	0.42	0.94	7.4	28.0
53107	5381	0.00	1.53	1.30	2.27	5.36	0.63	0.20	0.00	6.20	0.49	0.91	7.9	

53397	5420	0.30	0.11	0.90	2.15	0.12	0.03	0.01	0.00	0.17	0.41			28.2
54671	5506	0.21	0.63	1.00	1.34	0.32	0.04	0.02	0.00	0.38	0.48		7.5	28.4
55515	5532				2.70	0.22	0.41	0.00	0.00	0.62	0.42	0.89	7.7	28.2
56363	5527	0.00	0.00	0.10	0.30	0.08	0.02	0.00	0.00	0.11	0.54	0.87	7.4	20.1
58740	5727	0.21	0.24	1.50	0.71	0.33	0.05	0.00	0.01	0.39	0.62	0.79	8.0	28.7
60163	5766	0.37	0.56	1.20	0.60	0.31	0.06	0.00	0.00	0.36	0.58	0.92	7.0	26.2
61559	5891	0.00	0.00	0.20	0.73	0.01	0.01	0.00	0.00	0.01	0.40	0.85	7.3	29.4
64427	6058	0.00	0.12	0.40	2.63						0.39	0.93	7.5	29.0
65610	6188	0.37	0.86	1.50	1.64	0.46	0.13	0.01	0.01	0.61	0.46	0.91	7.2	28.8
67466	6273	0.50	3.71	2.60	9.32	1.26	0.42	0.04	0.00	1.72	0.31	0.89	7.6	27.5
68905	6448	0.06	0.22		3.48	0.04	0.01	0.00	0.00	0.05	0.32	0.96	7.6	26.8
71862	6753	0.29	1.27		9.87	0.24	0.05	0.02	0.00	0.31	0.23	0.87	7.8	29.3
73472	10	0.50	3.08	8.30	1.77	0.37	0.14	0.03	0.02	0.56	0.50	0.87	7.7	27.8
75382	7031	0.30	1.28	1.40	2.87	0.09	0.03	0.01	0.01	0.15	0.44	0.65	9.3	28.2
76835	7128	0.96	0.74	2.70	4.46	0.34	0.07	0.03	0.00	0.44	0.36	0.82	9.3	23.7
78300	7290	2.73	2.80	8.70	15.05	0.56	0.12	0.11	0.00	0.79	0.24	0.78	8.2	23.6

SI References

1. Blaauw, M. & Christen, J. A. Flexible paleoclimate age-depth models using an autoregressive gamma process. *Bayesian Anal.* **6**, 457–474 (2011).
2. Russell, J. M., Hopmans, E. C., Loomis, S. E., Liang, J. & Sinninghe Damsté, J. S. Distributions of 5- and 6-methyl branched glycerol dialkyl glycerol tetraethers (brGDGTs) in East African lake sediment: Effects of temperature, pH, and new lacustrine paleotemperature calibrations. *Org. Geochem.* **117**, 56–69 (2018).
3. Zhao, B. *et al.* Evaluating global temperature calibrations for lacustrine branched GDGTs: Seasonal variability, paleoclimate implications, and future directions. *Quat. Sci. Rev.* **310**, 108124 (2023).
4. Jaeschke, A. *et al.* Influence of land use on distribution of soil n-alkane δD and brGDGTs along an altitudinal transect in Ethiopia: Implications for (paleo)environmental studies. *Org. Geochem.* **124**, 77–87 (2018).
5. Peaple, M. D. *et al.* Identifying the drivers of GDGT distributions in alkaline soil profiles within the Serengeti ecosystem. *Org. Geochem.* **169**, 104433 (2022).
6. Bittner, L. *et al.* A Holocene temperature (brGDGT) record from Garba Guracha, a high-altitude lake in Ethiopia. *Biogeosciences* **19**, 5357–5374 (2022).
7. Wang, H. *et al.* Salinity-controlled isomerization of lacustrine brGDGTs impacts the associated MBT5ME’ terrestrial temperature index. *Geochim. Cosmochim. Acta* **305**, 33–48 (2021).
8. Kou, Q. *et al.* Distribution, potential sources, and response to water depth of archaeal tetraethers in Tibetan Plateau lake sediments. *Chem. Geol.* **601**, 120825 (2022).
9. Günther, F. *et al.* Distribution of bacterial and archaeal ether lipids in soils and surface sediments of Tibetan lakes: Implications for GDGT-based proxies in saline high mountain lakes. *Org. Geochem.* **67**, 19–30 (2014).
10. Li, J. *et al.* Distribution of glycerol dialkyl glycerol tetraether (GDGT) lipids in a hypersaline lake system. *Org. Geochem.* **99**, 113–124 (2016).
11. Martínez-Sosa, P. *et al.* GDGT-based determination of paleoenvironments via machine learning. in vol. 2021 1–2 (European Association of Geoscientists & Engineers, 2021).

12. Yao, Y. *et al.* Correlation between the ratio of 5-methyl hexamethylated to pentamethylated branched GDGTs (HP5) and water depth reflects redox variations in stratified lakes. *Org. Geochem.* **147**, 104076 (2020).
